# Applying the Theoretical Domains Framework to identify barriers and targeted interventions to enhance nurses’ use of electronic medication management systems in two Australian hospitals

**DOI:** 10.1186/s13012-017-0572-1

**Published:** 2017-03-27

**Authors:** Deborah Debono, Natalie Taylor, Wendy Lipworth, David Greenfield, Joanne Travaglia, Deborah Black, Jeffrey Braithwaite

**Affiliations:** 1 Centre for Healthcare Resilience and Implementation Science, Australian Institute of Health Innovation, Macquarie University, Sydney, NSW Australia; 2 Centre for Values, Ethics and Law in Medicine, University of Sydney, Sydney, NSW Australia; 3 Faculty of Health, University of Technology, Sydney, NSW Australia; 4 Faculty of Health Sciences, University of Sydney, Sydney, NSW Australia; 5 Australian Institute of Health Services Management, University of Tasmania, Sydney, Australia

**Keywords:** Implementation, Theoretical Domains Framework, Behaviour change, Electronic Medication Management Systems, Medication administration, Workarounds

## Abstract

**Background:**

Medication errors harm hospitalised patients and increase health care costs. Electronic Medication Management Systems (EMMS) have been shown to reduce medication errors. However, nurses do not always use EMMS as intended, largely because implementation of such patient safety strategies requires clinicians to change their existing practices, routines and behaviour. This study uses the Theoretical Domains Framework (TDF) to identify barriers and targeted interventions to enhance nurses’ appropriate use of EMMS in two Australian hospitals.

**Methods:**

This qualitative study draws on in-depth interviews with 19 acute care nurses who used EMMS. A convenience sampling approach was used. Nurses working on the study units (*N* = 6) in two hospitals were invited to participate if available during the data collection period. Interviews inductively explored nurses’ experiences of using EMMS (step 1). Data were analysed using the TDF to identify theory-derived barriers to nurses’ appropriate use of EMMS (step 2). Relevant behaviour change techniques (BCTs) were identified to overcome key barriers to using EMMS (step 3) followed by the identification of potential literature-informed targeted intervention strategies to operationalise the identified BCTs (step 4).

**Results:**

Barriers to nurses’ use of EMMS in acute care were represented by nine domains of the TDF. Two closely linked domains emerged as major barriers to EMMS use: Environmental Context and Resources (availability and properties of computers on wheels (COWs); technology characteristics; specific contexts; competing demands and time pressure) and Social/Professional Role and Identity (conflict between using EMMS appropriately and executing behaviours critical to nurses’ professional role and identity).

The study identified three potential BCTs to address the Environmental Context and Resources domain barrier: adding objects to the environment; restructuring the physical environment; and prompts and cues. Seven BCTs to address Social/Professional Role and Identity were identified: social process of encouragement; pressure or support; information about others’ approval; incompatible beliefs; identification of self as role model; framing/reframing; social comparison; and demonstration of behaviour. It proposes several targeted interventions to deliver these BCTs.

**Conclusions:**

The TDF provides a useful approach to identify barriers to nurses’ prescribed use of EMMS, and can inform the design of targeted theory-based interventions to improve EMMS implementation.

**Electronic supplementary material:**

The online version of this article (doi:10.1186/s13012-017-0572-1) contains supplementary material, which is available to authorized users.

## Background

Medication errors cause significant iatrogenic harm in hospitals worldwide [1–6] and are estimated to occur in 5–10% of in-hospital medication administrations [1]. In addition to harming patients, medication errors undermine patients’ confidence in the healthcare system, extend hospital length of stay and are costly [7–9].

Internationally, sustained efforts endeavour to reduce medication error rates [10]. One common approach has been the implementation of Electronic Medication Management Systems (EMMS) [11]. EMMS are designed to digitise administration processes, structure medication-related tasks, provide information support and promote adherence to medication administration policies [12].

Implementing EMMS has reduced errors in documentation [13] and prescribing and administration [14–17] and has improved adherence to safety guidelines [18, 19]. Yet, nurses do not always use EMMS as intended. Rather, nurses use ‘workarounds’—practices that differ from organisationally prescribed or intended procedures—to circumvent perceived or actual hindrances to achieving a goal [20]. EMMS workarounds include not taking an electronic medication administration record to the patient [21–23]; preparing medications for multiple patients concurrently [24]; entering medication as administered before having done so [23]; and signing off medication in the EMMS that has been administered by another nurse [22]. While there is little empirical evidence for the impact (negative or positive) of these workarounds on patient safety, not using EMMS appropriately undermines the potential to isolate and measure the impact of EMMS on medication error and may increase the potential for error [21, 25–28].

Given that some EMMS-related workarounds may be unsafe, it is important to understand the barriers associated with EMMS use. To date, examination of these barriers has focused largely on the mismatch between workflow and introduced technology, and on shortcomings in EMMS design, posing barriers to using computers in clinical settings [21, 22, 24, 26–32]. While useful, this research does not directly recognise that patient safety interventions, such as EMMS implementation, require healthcare professionals to change their behaviour. Theoretical approaches to identifying barriers to behaviour change and to designing targeted interventions to address them have been demonstrated to be more successful in changing behaviour than non-theory-driven approaches [33–36]. For example, one meta-analysis identified that studies that explicitly used theory for intervention design were significantly more effective in changing behaviour than interventions that did not [35]. To date, we have not identified any theory-driven behaviour change strategies that have tackled nurses' resistance to the use of EMMS. This research used the Theoretical Domains Framework (TDF) to address this gap.

The TDF is a systematic and theoretically based approach to behaviour change that is used to detect key barriers to changing practice and to devise practical interventions to counter them [37–41]. The TDF comprises 14 domains (Table 1) representing barriers comprising 84 theoretical constructs from multiple psychological and organisational behaviour change theories: Knowledge, Skills, Social/Professional Role and Identity; Beliefs About Capabilities; Optimism; Beliefs about Consequences; Reinforcement; Intentions; Goals; Memory, Attention and Decision Processes; Environmental Context and Resources; Social Influences; Emotion; and Behavioural Regulation [39].

**Table 1:** Definitions of the theoretical domains [37] (Definitions are based on definitions from the American Psychological Associations’ Dictionary of Psychology [75])

Theoretical domain	Definition
Knowledge	An awareness of the existence of something
Skills	An ability or proficiency acquired through practice
Social/professional role and identity	A coherent set of behaviors and displayed personal qualities of an individual in a social or work setting
Beliefs about capabilities	Acceptance of the truth, reality, or validity about an ability, talent, or facility that a person can put to constructive use
Optimism	The confidence that things will happen for the best or that desired goals will be attained
Beliefs about consequences	Acceptance of the truth, reality, or validity about outcomes of a behavior in a given situation
Reinforcement	Increasing the probability of a response by arranging a dependent relationship, or contingency, between the response and a given stimulus
Intentions	A conscious decision to perform a behavior or a resolve to act in a certain way
Goals	Mental representations of outcomes or end states that an individual wants to achieve
Memory, attention and decision processes	The ability to retain information, focus selectively on aspects of the environment and choose between two or more alternatives
Environmental context and resources	Any circumstance of a person’s situation or environment that discourages or encourages the development of skills and abilities, independence, social competence, and adaptive behavior
Social influences	Those interpersonal processes that can cause individuals to change their thoughts, feelings, or behaviours
Emotion	A complex reaction pattern, involving experiential, behavioral, and physiological elements, by which the individual attempts to deal with a personally significant matter or event
Behavioral regulation	Anything aimed at managing or changing objectively observed or measured actions

The theoretical domains have been mapped to specific behaviour change techniques (BCTs) [37, 42], which are the active components of interventions related to each domain [43]. A taxonomy of BCTs, linked to theoretical constructs associated with changes in specific behaviours [37, 42], can generate targeted remedial interventions to elicit behaviour change and improve patient outcomes (e.g. [44, 45]).

The TDF has been used to identify barriers to implementing a range of practices with implications for patient safety including reporting adverse drug events in hospitals [46], reducing prescribing errors among trainee doctors [47], preventing misplaced nasogastric feeding tubes [44, 45], implementing stroke guideline recommendations [48], and encouraging hand hygiene practices [33].

This study focuses on barriers to nurses’ appropriate use of EMMS, because nurses are the predominant users of EMMS to prepare and administer medication in acute care settings. It used the TDF to characterise emergent barriers to nurses’ appropriate use of EMMS, to identify the most relevant (pre-defined) BCTs that have been mapped to specific domain barriers [37, 42] from the literature and to propose targeted interventions that could potentially address barriers [37, 39, 42, 43, 49]. It therefore fills an identified gap in theory-based interventions to address medication error [50]. Appropriate use of EMMS is anticipated to improve patient safety through reduction of medication error.

## Methods

**Fig. 1:**
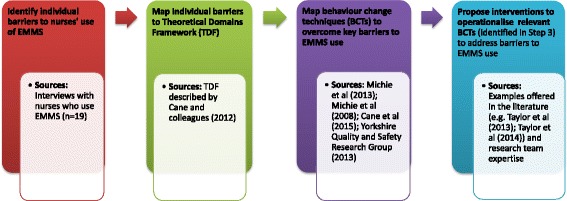
Overview of study design. This study comprises four steps: interviews to explore nurses’ experience of using EMMS and identification of barriers to doing (step 1); analysis of data using TDF to identify barriers to appropriate use of EMMS (step 2); identification of relevant behaviour change techniques (BCTs) to address key barriers to using EMMS (step 3); and identification of potential targeted intervention strategies to operationalise the identified BCTs (step 4)

This qualitative study presents data from interviews conducted with 19 nurses. It was part of a larger ethnographic study investigating nurses’ use of EMMS in everyday practice [51]. The current study followed four steps (Fig. 1). The qualitative approach enabled researchers to explore, explain and describe complex processes and behaviours within the context in which they occur [52, 53].

### Step 1: collecting data using semi-structured interviews

#### Setting

At the time of the study, a statewide phased implementation of IT, including medication management, had been recommended for New South Wales (NSW) acute care hospitals [54, 55], but this was not yet fully realised. Data were collected at two large metropolitan university-affiliated teaching hospitals (over 300 beds) in NSW, Australia (henceforth, hospital 1 (H1) and hospital 2 (H2)), that had commenced staged rollout of EMMS approximately six years before data collection. At each hospital, the sample included three units that used different EMMS and models of nursing care to maximise variation [56].

#### Sampling and study participants

Given the ever-changing demands across nurses’ shifts, a convenience sampling strategy was used. Nurses who used EMMS were opportunistically invited by the researcher to participate if they were available when interviews were being conducted; one nurse declined to participate. This sampling approach allowed for the inclusion of nurses with varying experience using EMMS [52, 57]. Participants were allocated a randomly selected unique identification number.

#### Interview procedure

Team members with extensive health services research experience collaboratively developed the topic guide that directed semi-structured interviews and prompted more detailed discussion led by the participants. DD conducted face-to-face interviews at times to most suited participants. Interviews ranged between 18 and 89 min duration (mean = 35 min; median = 31 min). Open-ended questions facilitated exploration of nurses’ experiences using EMMS that were salient to the interviewees (Table 2). Questions encouraged participants to relate their knowledge and reflect on their experience of using EMMS. Interviews analysed in this study were digitally recorded and later transcribed^1^.

**Table 2:** Interview questions

Could you explain the electronic medication management system that is used in this unit to me please?
Can you tell me about the medication process that is used in this unit? [Prompts: Is there a medication round, nurse dedicated to medication delivery, pharmacy round etc]?
Can you tell me about how has using the electronic medication management system changed aspects of your work?
Are there times when it is difficult to use the electronic system in administering medication? Can you tell me about some of the things that make it difficult?
Can you tell me about what do you do when something makes it difficult to get the medication to the patient?
Does everyone use the same practices to get the medication to the patient? Can you tell me about how the practices differ between nurses?
Can you tell me about whether and how you workaround the system to get the medication to the patient?
Can you tell me about whether and how other people workaround the system the system to get the medication to the patient?
Would you explain for me if there are times when it is OK to workaround the system to get the medication to the patient and when it is not OK ? Is this the same for everyone?
Can you tell me about times when it is OK for some nurses to workaround the system the system to get the medication to the patient but not OK for others to workaround?
Are there times when it is easier to use the electronic system in administering medication? Can you tell me about some of the things that make using the electronic medication management system easier?
Can you tell me what impact you think the electronic medication management system has had on quality and safety?
What sort of things impact on the use of the electronic medication management system? [For example, experience with the system, business of the shift, staff levels]

### Step 2: using the TDF to identify key barriers to implementation of EMMS

**Fig. 2:**
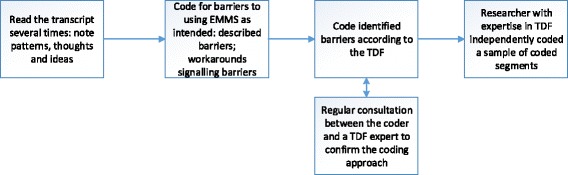
Overview of analysis process. Data were analysed across three stages: Interview transcripts were randomly selected for analysis from each unit. Stage 1: The first author read interview transcripts, randomly selected from each study ward. Stage 2: Transcripts were interrogated at a descriptive level for barriers to using Electronic Medication Management Systems (EMMS). Stage 3: Participants’ statements about barriers to EMMS use were coded into 14 conceptual domains comprising the Theoretical Domains Framework. Individual barriers were tabulated by domain. A second coder with extensive experience using the TDF independently coded a sample of data segments. Discrepancies were resolved through discussion

Data analysis began with inductive coding for barriers that arose from the data, followed by grouping identified barriers into categories based on the TDF. Data were analysed across three stages when all data had been collected (Fig. 2). Stage 1: Interview transcripts were randomly selected for analysis from each unit. The first author read the transcripts several times noting patterns, thoughts and ideas. Stage 2: initial/open coding: Transcripts were interrogated at a descriptive level [58] for statements indicating barriers to using EMMS. Segments of text highlighting barriers to appropriate use of EMMS were copied and pasted into an Excel file. Stage 3: Participants’ statements about barriers to EMMS use were coded into 14 conceptual domains comprising the TDF, described by Cane and colleagues [39]. Statements were assigned to a relevant TDF domain or set of domains. Selection of the most appropriate barrier domain or domains was based on the topic and context of the statement within the entire interview. Individual barriers were tabulated by domain. To increase reliability of the assignment of barriers to a relevant TDF domain, a second coder (NT) with extensive experience using the TDF [40, 44, 45, 59] independently coded a sample of data segments until we were confident that there was agreement (approx. 9% of coded data). The second coder was provided with coded segments accompanied by a summary explanation to provide meaning in context of the full interview (examples in Additional file 1: Table S6 and Additional file 2: S7). Discrepancies were resolved through discussion. Thematic saturation, when no new TDF-related themes were emerging from the analysis [60], occurred when 19 interviews had been coded (Table 3).

**Table 3:** Number of interviews coded using TDF by unit

Hospital/unit	Number of interviews coded using TDF
Hospital 1/1	4
Hospital 1/2	4
Hospital 1/3	4
Hospital 2/1	3
Hospital 2/2	2
Hospital 2/3	2

### Step 3: identifying appropriate BCTs to address barriers to EMMS use as intended

To identify potential BCTs to address the barriers identified in step 2, we drew upon literature that maps particular BCTs to the behavioural determinants (domains) they are effective in changing [37, 42, 49]. We selected BCT groupings from Michie et al.’s BCT Taxonomy (v1) of 93 hierarchically clustered techniques [43]. We then selected individual BCTs that appeared to address key barriers to nurses’ use of EMMS and that would be easiest to operationalise in a given context. To illustrate, *Identification of self as role model*, within the BTC taxonomy grouping *Identity*, can be an effective BCT to address barriers related to the Social/Professional Role and Identity domain.

### Step 4: generating potential intervention strategies to address barriers to appropriate use of EMMS

Where possible, we used successful examples offered in the literature [44, 45, 49, 61, 62], and the expertise of the research team, to propose interventions to operationalise the relevant BCTs identified in step 3.

### Validity

To ensure validity and credibility, we double-coded as described above, frequently debriefed with the research team and member checked [63] with participants during feedback sessions in 2014 and 2015. Nurses indicated their agreement with the identified barriers, in some cases offering additional examples or counter-examples.

### Ethics approval

Ethics approval was granted by a health service Human Research Ethics Committee (HREC) and ratified by a University HREC (approval number: HC09223 ). Participants provided written informed consent.

## Results

Participants (10 females and nine males) included senior and newly graduated registered nurses and enrolled nurses who were endorsed to administer medications (Table 4). Participants’ experience in their current role ranged between less than 1 year and more than 10 years (Table 4).

**Table 4:** Role and experience of participants

Role (years in role)	Number of participants
Registered nurse (more than 10 years)	8
Registered nurse (1–10 years)	6
Newly graduated nurse (less than 1 year)	2
Endorsed enrolled nurse (3–10 years)	3

### Medication management and EMMS

At both hospitals, medications were stored in a medication room when they required injection, refrigeration or co-signature for administration. Other prescribed oral medications were stored in each patient’s bedside cabinet, in a locked drawer to which nurses had a key.

**Fig. 3:**
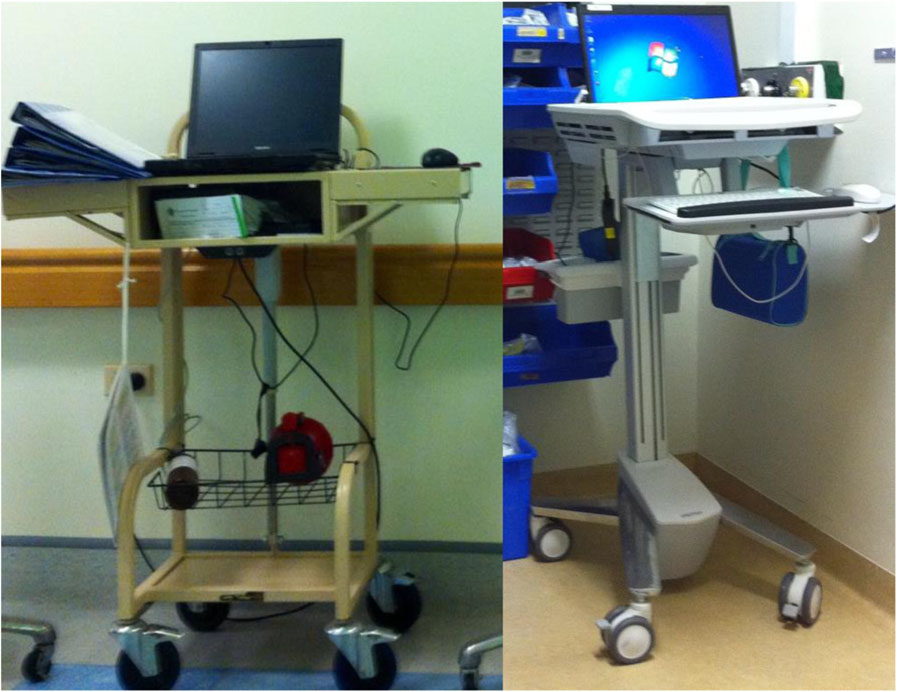
Computers on wheels (COWs)

While the design and user interface of the EMMS differed between the hospitals, both systems included ePrescribing, pharmacy review and medication administration. At both hospitals, the EMMS comprised the electronic medication administration record (eMAR), a permanent and legal record of the medications prescribed and administered to patients, and of who prescribed and administered them. Computers on wheels (COWs; Fig. 3) enabled access to clinical and medication information at point-of-care. Features of the EMMS at both hospitals had been enhanced since implementation.

Nurses at both hospitals logged into the EMMS electronically and opened a patient’s eMAR. They recorded medication administration as ‘successful’, ‘withheld’, ‘delayed’ or ‘not given’ and then nurses logged out of the EMMS. Table 5 summarises 19 key behaviours involved in administering medication using EMMS.

**Table 5:** Key behaviours involved in using EMMS to administer medication

Behaviour ID	Key behaviour
B1	Nurses endorsed to do so use the EMMS to administer medication.
B2	The *administering* nurse logs into the EMMS and opens the patient’s eMAR.
B3	When leaving the eMAR, the administering nurse logs off the eMAR or changes user.
B4	Open a single patient’s eMAR at a time. With only their eMAR open prepare medication for one patient at a time immediately before intended use (includes medications requiring a second person check or witness).
B5	Check the eMAR to identify when medications are due.
B6	Check the eMAR to ascertain when the medication was previously administered.
B7	Within scope of practice limitations nurse select medications in the eMAR.
B8	Check medication preparation and administration instructions and alerts/icons in the eMAR.
B9	Check the purpose, action and safe dose range of the medication to be administered prior to administering the medication.
B10	Complete associated medication-related tasks and enter information in the eMAR prior to administering medication (e.g. blood glucose level, blood pressure, heart rate).
B11	Check medication-associated test results (e.g. electrolyte levels) on the system prior to administering medication.
B12	Take an eMAR to the patient to check the 5 Rights of medication administration prior to administration of a medication. Check the 5 Rights of medication administration against the patient’s eMAR and the patient prior to administering medication.
B13	Ascertain that the patient is not allergic to the medication to be administered by checking the patient’s allergies listed in the eMAR and with the patient prior to administering the medication. This requires that a responsive eMAR be taken to the patient when administering medication.
B14	Medications requiring a witness: a responsive eMAR must be taken to the patient to check the 5 Rights of medication administration prior to administration of a medication. Check the 5 Rights of medication administration against the patient’s eMAR and the patient prior to administering medication. In addition, preparation, administration and discarding of unused medications must be witnessed and the witness must enter their username and password in the eMAR following medication administration and discarding of unused medication (at the time of medication administration).
B15	Medications requiring a second person check: a responsive eMAR must be taken to the patient to check the 5 Rights of medication administration prior to administration of a medication. The administering and checking nurse check the 5 Rs verifying the patient identification, allergies and order in the eMAR. The administering nurse is logged into the eMAR, and the details of the checking nurse should be recorded in the eMAR at the time of medication administration.
B16	Assess whether it is safe to administer medication (is there any reason why the medication should *not* be administered (e.g. oral medication when patient is unconscious; administration of medication is contraindicated on the basis of tests, fasting, etc).
B17	If appropriate (see B16), administer medications according to a time frame prescribed in the eMAR.
B18	Relevant information about medication administration is recorded and communicated. Medication administration is to be recorded in the eMAR by the administering nurse once it has successfully been administered (at the time of administration). Oral medication must be observed to be consumed by the patient before the administering nurse enters it as administered in the eMAR. Unadministered medication should be recorded accordingly—‘not given’, ‘withheld’, ‘rescheduled’, ‘delayed’ and a reason entered. If a variable medication dose is ordered, the dose administered should be recorded in the eMAR. If the administered dose is different from the prescribed dose, the amount administered and the reason for the difference should be entered in the eMAR.
B19	When the administration information has been entered, the screen should be refreshed/the eMAR closed.

### Barriers to appropriate use of EMMS

Nine domains of the TDF collectively captured the major barriers to nurses’ use of EMMS: Environmental Context and Resources; Social/Professional Role and Identity; Knowledge; Beliefs about Consequences; Beliefs about Capabilities; Social Influences; Memory, Attention and Decision Processes; Emotion; and Intention. Additional file 1: Table S6 contains descriptions of these domains, behaviours for which they are a barrier, and related evidence from the interviews. Some barriers could be attributed to more than one domain depending on the context.

Of these nine domains, two were more richly described than the others: Environmental Context and Resources and Social/Professional Role and Identity. We include illustrative data excerpts from the manuscript to highlight barriers relevant to these two domains. Further evidence is provided in (Additional file 2: Table S7) in the form TableNumber_QuoteNumber (e.g. T7_Q1).

We then identify BCTs that have been shown to be effective in influencing these two theoretical domains. For each barrier, we present an example of a BCT operationalised into a potential context-specific intervention strategy, italicised in brackets (see Additional file 2: Table S7).

### Environmental Context and Resources

According to the TDF, available resources and the environment in which a behaviour must be performed influence a person’s inclination to perform it [40]. Environmental Context and Resources emerged as a key barrier to appropriate use of EMMS and is frequently affected by availability and properties of COWs; technology characteristics; specific contexts; and competing demands and time pressure.

### Availability and properties of COWs

Unavailability of COWs represented a key barrier to nurses taking the eMAR to a patient when administering medication. During busy times (e.g. morning medication rounds), there were insufficient COWs available for every nurse (T7_Q1). Increased competition for COWs occurred when doctors’ ward rounds coincided with nurses’ medication rounds.

If we don’t have a laptop for every nurse that’s on, that’s the big impact. There’s always one in the morning that doesn’t get the computer. (Interview 91)

Physical properties of the COWs also made their use difficult. Nurses complained about dimming screens, short battery life, difficulty adjusting the trolley height and computer deterioration. The COWs were hard to clean, heavy to push and challenging to manoeuvre. When nurses judged that adding equipment to already crowded rooms created a fall risk, they did not take the COW to the bedside to administer medication (T7_Q2).

If they find out it’s too much equipment, too many furnishings in the room and it’s high risk for a fall for the patients, they can leave it outside and get the drawer. Just take the single drawer, put it on the COW and dispense the medication, put it back, check their MRN number and go to the patient and give it. (Interview 42)

At night, the noise of the COWs, the risk of bumping into things and the bright screens were likely to wake sleeping patients or agitate confused patients. Nurses worked around this by not taking a COW to their bedsides (T7_3).

Specific contexts, such as when patients were isolated, presented additional barriers. Infection control policies required equipment to be left in the isolation room or to be cleaned down when being removed. There were insufficient COWs to dedicate to isolation rooms, and their physical properties made them difficult and time-consuming to disinfect (T7_Q4).

### Possible targeted interventions

Increasing the number of COWs (BCT: *adding objects to the environment*) and introducing handheld devices that can be easily cleaned or encased in disposable covering for use with isolated patients (BCT: *restructuring the physical environment*).

### Features of the EMMS

At H1, the EMMS had a short automatic logout time. Information that had been entered but not saved until that point had to be re-entered. If nurses waited to sign off medication until after administration, they risked being logged off prematurely (T7_Q5).

It only lasts a while before it logs out so you can’t be taking someone to the toilet or whatever, it’ll log out and you’ve got to log back in and you’ll have lost everything (Interview 3)

At H2, where several staff could be active in the eMAR concurrently, medication orders could be changed while nurses were administering medication (T7_Q6) and could not be recorded in eMAR. If the order was replaced with an updated order for the same medication, there was also potential for another nurse to administer the same medication from the updated order if there was no record of the medication being previously administered. At both hospitals, nurses signed off medication before, rather than after; it had been administered to circumvent these technology-related barriers.

the doctor ceased the medication on the other terminal. I was giving out the medication and gave it to the patient, was going to sign the order and then find out the order is not there anymore. (Interview 30)

Default medication administration times that did not match local context, such as meal times, were a barrier to administering medication at the prescribed time. Nurses either changed the medication times in the eMAR (additional steps) or administered medication and signed it off in the eMAR later when it became ‘available for administration’ (T7_Q7).

Sometimes with the paper charts, if the times weren’t suitable or something needed to be given with meals, we just changed the times ourselves or even ceased drugs if you knew they were just for 48 h.

[...]

I'll sometimes give it at the - what I'd think is the correct time and then sign it later and maybe change it later on. (Interview 57)

### Possible targeted interventions

Lengthening logout time (H1), introducing an automatic screen lock without logout, restructuring the EMMS to allow one authorised user at a time (H2) (BCT: *restructuring the physical environment*), and introducing an alert in eMAR or providing stickers on computers to remind doctors to consider local context when prescribing (e.g. ‘06:30 is 1 h before breakfast on Unit X’) (BCT: *prompts*/*cues*) [61].

### Time pressure and competing demands

Nurses frequently juggled competing demands, heavy workloads and insufficient resources to complete tasks (e.g. observations, medications, showers and dressings) within specific time frames (e.g. doctors’ rounds, appointments, meal times and shift times). Nurses admitted that when really busy, they did not always use the EMMS appropriately—e.g. not taking the COW to the bedside (T7_Q8) or not witnessing medication administration by two nurses when required.

If you’re in a rush and sometimes you just can’t—it’s more accessible for you just to do your stuff at the computer, run to the patient and run back (Interview 39)

Time pressure was exacerbated by limited staff authorised to use EMMS and the timing of medication rounds. In one unit, where the medication round had been rescheduled, nurses described a reduction in competing demands.

### Possible targeted interventions

Ensuring there are sufficient resources, including staff authorised to administer and check medication by either changing the times of ward rounds or personal care or adding staff to the medication administration process (e.g. appointing one nurse to have the role of checking/witnessing medications) (BCT: *adding objects to the environment*) [45]).

### Social/Professional Role and Identity

According to the TDF, the degree to which a behaviour is believed to align with, strengthen or undermine a person’s social or professional role and identity will influence whether they will implement the behaviour [40]. Nurses’ appropriate use of EMMS was strongly influenced by whether it supported or challenged their Social/Professional Role and Identity.

Using the EMMS appropriately hindered nurses from effectively executing behaviours that were an important part of their professional role and identity, including having the authority to administer medications, being time-efficient, considering patient preferences, promoting patient safety and demonstrating respect for colleagues.

### Having the authority to administer medications

The EMMS blocked nurses without specific authorisation from administering medication, resulting in anger and frustration (T7_Q9) or a sense of being relegated to ‘basic nursing tasks’, hampering their ability to fully care for their patients and help their colleagues. This led to non-adherence to policy; in some circumstances, colleagues signed off administration of medication for those who could not (T7_Q10).

that affects their routine then, because they’re then waiting for us to come and do something for them that might be stopping them from doing something else. So it holds them back in their patient care. Most of the EENs here will—are happy to—once they’ve had it checked by one of us, they’re happy to administer it. (Interview 61)

### Possible targeted interventions

Visual images to convey approval of nursing professional bodies, lawyers and managers to adherence to sign off requirements in the EMMS, for example posters of opinion leaders saying *Sign it only if you administered it* positioned in places where nurses prepare medications (BCT: *information about others*’ *approval*).

### Being time-efficient

Being time-efficient was important to nurses’ professional role and identity. Therefore, when using the EMMS which appropriately slowed nurses down, it did not support their professional role and identity. Participants described cutting corners such as administering medication earlier than prescribed (T7_Q11) and not taking the COW to the bedside in order to save time.

I think even the juniors, they’re a lot more anxious they want to get it done before anyone else, before anyone else has to check on them (Interview 39)

Overdue medication alerts (OMA) posed a particularly strong threat to nurses’ identities as efficient professionals in some units. Negative reactions included anxiety, frustration and a perception of failure to perform their role adequately. Responses to OMAs appeared to differ between units and hospitals. Some nurses described rushing to avoid the OMA (T7_Q12) or entering ‘delay’ to remove the OMA from the computer screen, while others identified the OMA as a useful reminder that medication is needed to be administered.

Like on a busy morning shift, 9 o’clock you’re only up to two patients and there are four patients with alarm clock next to it and you feel like a sense of failure maybe … you’re slower than the others. Yeah like you’re no good, you’ve got poor time management. (Interview 31)

### Possible targeted interventions

Persuasive communication during professional development sessions to draw attention to the discrepancies between nurses’ practice of rushing and administering medication earlier than prescribed and their self-image as a nurse who takes the time to administer medication safely (BCT: *incompatible beliefs*) and facilitated workshops to challenge the view that OMAs reflect poor practice—particularly where they are used as a reminder that medication is yet to be administered [62] (BCT: *social comparison*).

### Delivering patient-centred care

An important component of a nurse’s role is to deliver care that considers patients’ individual needs. A potential barrier to use of EMMS appropriately was, therefore, the perception that doing so would impede providing patient-centred care. Nurses did not, for example, want to take the COW to the bedside when it interrupted patients’ sleep or increased agitation (T7_Q3). Participants reported EMMS features that made it difficult to administer medication when patients requested it or when nurses judged it clinically appropriate (T7_Q13).

Sometimes when patients want Panadol early, sometimes it won’t be available in the system but you want to give it, so sometimes you just give it, then go in later and click it off. (Interview 91)

### Possible targeted intervention

Introducing professional development sessions to present a persuasive message emphasising that the EMMS aims to protect patients from medication error and staff from legal retribution, and is just as important as other dimensions of patient-centred care (BCT: *framing*/*reframing*) [62].

### Delivering safe care

A core nursing competency is to prevent injury by identifying, eliminating or preventing environmental hazards where possible. A key barrier to use of EMMS as intended was, therefore, the perception that to do so would place patients or others at risk. Nurses reported leaving EMMS outside patients’ rooms to diminish the risk of falls or cross-infection. Taking the COW to the bedside (using EMMS appropriately) did not align with professional role and identity when patients were isolated for infection control purposes, and nurses left COWs outside an isolated patient’s room in order to prevent cross-infection (T7_Q2 and T7_Q4). Nurses also identified the potential for interruptions and subsequent risk of medication error to be a barrier to taking the COW to the patient to administer medication (T7_Q15). To minimise interruptions, nurses prepared medications away from the bedside (T7_Q14).

But the more I’m at the bedside, patients start asking questions, and that’s kind of when you lose your thoughts. So I’d rather look at the doses in the drug room where it’s quiet, rather than at the bedside where other patients are … (Interview 91)

### Possible targeted interventions

Prompting nurses to deliberately adopt a new perspective on the benefits of using the EMMS appropriately (BCT: *framing*/*reframing*), e.g. indicating that taking the COW into an isolated room saves time by providing easy access to information about tests and point-of-care information, and improving legibility of medication orders or providing other options for cleaning the COW efficiently; demonstrating how to manage interruptions during medication administration (BCT: *demonstration of behaviour*) may be done face-to-face during group professional development sessions or by using a video.

### Respecting colleagues

Collegiality and teamwork were considered important to nursing professional behaviour. The professional importance nurses placed on not impinging on their colleagues’ time was therefore a barrier to using the EMMS appropriately. Rather than ask a colleague to check medications for one patient at a time, nurses therefore prepared and signed off medications for multiple patients in the EMMS when a colleague was available (T7_Q16).

Nurses did not consider that it was consistent with their position to log doctors and pharmacists out of the eMAR. Rather than log a colleague out or reclaim the COW they were using, nurses used desktop computers to administer medication and to protect against losing information if they were logged out. Alternatively, nurses signed off medication in the eMAR as having administered it to the patient before doing so (T7_Q17).

### Possible targeted interventions

Using persuasive communication to draw attention to the discrepancies between safe medication administration (part of the image of an ideal nurse) and signing medication as administered before doing so (BCT: *incompatible beliefs*), highlighting the potential implications of a medication error for the patient, and the administering and checking nurses or demonstrating to nurses how to discuss with colleagues the impact of logging them off the eMAR and taking the COW when they are doing medication administration (BCT: *demonstration of the behaviour*).

### Professional culture

Nurses described a professional culture in some units where reporting problems was not considered a collective responsibility. Therefore, rather than report a problem with hardware or software to be fixed, nurses left broken equipment to one side, reducing the overall number of working COWs available. When there were not enough available laptops, nurses were more likely to use the desktop computers (T7_Q18).

We find it difficult keeping them maintained. People a) don’t take responsibility for them so won’t initiate if they notice something wrong with it, if it is physically broken or if it is a software problem they won’t initiate it, it will just be left in the corridor to the side so it decreases the number of resources. (Interview 65)

Participants also reported a professional culture where it was acceptable, particularly for senior nurses, not to take the COW to the patient to administer medication. The professional hierarchy made it difficult for junior nurses to ask senior nurses to follow policy (T7_Q19).

Yeah, and you’re setting, it’s out of your place, if you’re younger, to say, ‘Look aren’t you going to take [the COW] with you?’ (Interview 39)

### Possible targeted interventions

Using posters with pictures of senior members of staff, opinion leaders and patients to advocate nurses’ professional responsibility to their patients and team members to report broken equipment to ensure availability of working laptops for medication administration (BCT: *information about others*’ *approval*), displaying information on how to report problems with equipment and computers, reminding senior nurses that their medication administration behaviour is a role model for others, encouraging them to identify the importance of their actions in shaping their unit’s professional culture and inviting them to present information about medication administration as a part of ongoing education to encourage them to identify as role models (BCT: *identification of self as role model*).

## Discussion

Consistent with the literature on nurses’ use of EMMS, we identified several forms of non-compliance and ‘working around’ in response to perceived barriers to using EMMS [21–23]. More theory-driven implementation and evaluation of patient safety practice interventions have been called for [64, 65]. To our knowledge, this was the first study using the TDF to identify key barriers to nurses’ use of EMMS and to formulate targeted, theoretically based interventions to facilitate improvements. Applying the tested TDF approach, for the first time, to examine barriers to appropriate use of EMMS within the Australian healthcare system adds to the growing body of evidence for the utility of the TDF.

Our study found that nine (overlapping) TDF domains represented barriers to nurses’ appropriate use of EMMS, including two which were particularly richly described. Environmental context and resources proved key barriers to nurses’ appropriate use of EMMS. Other studies have highlighted barriers such as design shortcomings [21, 22, 26–28, 30, 66], time pressure and competing demands. The availability, size and characteristics of COWs and log-in time influenced nurses’ likelihood of taking them to the bedside, leaving unattended COWs logged in, or signing off medications before administration.

Proposed theoretically informed interventions support non-theoretically generated interventions previously offered to address specific features and contextual configuration barriers to EMMS use [32, 67]. Targeted interventions might include introducing handheld devices that can be easily used with isolated patients. It is crucial that ergonomic design improvements are based on assessment of workflow and environment and pilot-tested prior to implementation [29, 32]. McLeod and colleagues reported, for example, nurses preferred a smaller tablet over a COW but found that because the tablet was too small, medications were signed off at the desktop [22]. Previous findings highlight the benefits of point-of-care access to information about test results, medication administration instructions and patient information [68, 69]. These features should all be preserved on smaller devices.

Our results also highlight the role of social/professional identity as a key barrier to implementing EMMS. The introduction of technology changes nursing practices, processes, patient care and the meaning of nursing work [70–72], potentially undermining nurses’ confidence and threatening professional identity [20]. Nurses’ professional identity includes administering medication and efficiently delivering safe care, where possible, according to patient’s requests and needs. Behaviour change interventions may require nurses to prioritise one over another. Future studies should explore moderating variables influencing nurses’ judgements about using EMMS as intended.

### Implications for implementation

Implementation of these theoretically derived interventions requires consideration of location-specific relevance, feasibility and leadership. One strategy is to encourage a culture that facilitates staff-management trust to facilitate reporting and rectification of barriers.

Measures in other contexts have attempted to build upon the TDF in order to facilitate the development and implementation of strategies. The Theoretical Domains Framework Implementation (TDFI) approach has six steps to implementing interventions and has demonstrated efficacy in co-designing, with frontline clinicians and interventions to address local factors to affecting behaviour change [44, 45]. Applying the TDFI in the EMMS context would incorporate co-design, implementation and evaluation of intervention strategies to improve EMMS use.

One challenge in operationalising the TDF is mapping individual barriers to a single theoretical domain [48] and dealing with inevitable overlap between domains [41]. Barriers may fall into different TDF domains. For example, administering medications at prescribed times did not match with meal times (environmental context and resources), were considered to be not in patient’s best interests (beliefs about consequences), were not when the patient wanted to take medication (social/professional roles and identity) or should not be changed by nurses (social/professional roles and identity). Identifying which barriers and appropriate BCTs to target them requires an understanding of local context. This underlines the importance of involving healthcare professionals in identifying and interpreting barriers and co-designing specific interventions.

However, overlap between domains can provide practical advantages. Understanding the many facets to what initially appears to be a single barrier domain (e.g. difficulties cleaning COWs) allows for a multipronged approach for which the effect of different strategies may be synergistic. Theory-based interventions incorporating multiple BCTs and modes of delivery have a greater effect than those that do not [35, 73]. It is vital to ensure that the reports of these interventions are transparent and replicable to enhance understanding of change mechanisms.

### Study limitations, strengths and future directions

Interview data were coded retrospectively using the TDF approach. While this allowed for identification of the issues that are most salient to practising nurses, using the TDF to inform questionnaire design may have elicited reports of barriers that are not spontaneously reported [33]. Designing an interview schedule based on a theoretical framework could, therefore, supplement future studies of barriers to appropriate use of EMMS.

To enhance generalisability, studies examining healthcare professionals’ use of technology must consider its use across contexts [74]. A strength of this study was its sampling from two hospitals, multiple units and different types of EMMS, maximising variation and applicability of findings to other contexts. However, the participants were all nurses, therefore limiting the generalisability of study findings to other professions. This is particularly important given the relevance of the Social/Professional Role and Identity domain for nurses as a key barrier to EMMS implementation.

Nurses’ ability to adapt and balance competing demands during medication rounds has been documented [22]. Nurses in this study employed numerous workarounds to circumvent barriers in order to administer medication. Future work could usefully map these intuitively developed strategies to BCTs. Do they, for example, address some barriers to behaviour change while concurrently creating barriers to others? Do all nurses use workarounds to address barriers to performing the required practice or do certain factors moderate when they are implemented and by whom? Understanding how intuitively derived interventions for addressing key problems align with theory-based BCTs and barriers could further advance the science of implementation [65].

## Conclusions

This study identified barriers to the use of EMMS in daily practice to examine potential solutions to these barriers. Garnering the perspectives of nurses was an essential component of this process given their role in medication administration. The study demonstrated that the TDF provided a useful framework both to categorise and assess barriers to nurses’ appropriate use of EMMS and to suggest theory-based interventions to target these barriers.

## Additional files

Additional file 1: Table S6. Barrier examples and illustrative quotes by domain. Nine domains of the TDF collectively captured the major barriers to nurses’ use of EMMS: Environmental Context and Resources; Social/Professional Role and Identity; Knowledge; Beliefs about Consequences; Beliefs about Capabilities; Social Influences; Memory, Attention and Decision Processes; Emotion; and Intention domains. Descriptions of the nine domains, the component behaviours for which they are a barrier and evidence related to each from the interviews are provided in Table S6. (DOCX 49 kb)

Additional file 2: Table S7. Proposed interventions (modes of delivery of BCTs) mapped to barriers to EMMS use. Two domains of the TDF emerged more strongly than the others in capturing barriers to nurses’ use of EMMS: Environmental Context and Resources, and Social/Professional Role and Identity. In-text references to specific examples (Additional file 2: Table S7) are given in the form TableNumber_QuoteNumber (e.g. T7_Q1)]. We then identify BCTs that have been shown to be effective in influencing these two theoretical domains. For each barrier, we present an example of a BCT operationalised into a potential context-specific intervention strategy. To distinguish them from the specific targeted interventions designed to deliver them, BCTs are italicised in brackets. Additional examples are provided in (Additional file 2: Table S7). (DOCX 56 kb)

## Abbreviations

BCT: Behaviour change technique
COWs: Computers on wheels
eMAR: Electronic medication administration record
EMMS: Electronic medication management systems
NSW: New South Wales
OMAs: Overdue medication alerts
TDF: Theoretical Domains Framework
TDFI: Theoretical Domains Framework Implementation

## Footnotes

1 When presenting raw data as illustrative quotes, they have been minimally edited to remove filler words such as ‘um’, ‘ah’, ‘mm’ that do not add meaning to the content and synonyms used for words that, because they were particular to individuals, potentially identified them.
