# Supplementary material for: Applying the Theoretical Domains Framework to identify barriers and targeted interventions to enhance nurses’ use of electronic medication management systems in two Australian hospitals
Source: Implement Sci. 2017 Mar 27;12:42. doi: 10.1186/s13012-017-0572-1 (PMC5368903; doi:10.1186/s13012-017-0572-1)
Supplement: Supplementary file 1 — Barrier examples and illustrative quotes by domain. Nine domains of the TDF collectively captured the major barriers to nurses’ use of EMMS: Environmental Context and Resources; Social/Professional Role and Identity; Knowledge; Beliefs about Consequences; Beliefs about Capabilities; Social Influences; Memory, Attention and Decision Processes; Emotion; and Intention domains. Descriptions of the nine domains, the component behaviours for which they are a barrier and evidence related to each from the interviews are provided in Table S6. (DOCX 49 kb) [file 13012_2017_572_MOESM1_ESM.docx]

**Additional file 1: Table S6. Barrier examples and illustrative quotes by domain**

| **Barrier label** | **Barrier Description**  **(Specific behaviour)** | **Corresponding behaviour ID (Table 5)** | **[In text quote number] *Example quotes***  **Summary explanation** |
| --- | --- | --- | --- |
| **Environmental Context and Resources** | Unavailability of COWs was a barrier to taking an active eMAR to the patient when administering medication. | Take an active eMAR to the patient (B12) (B12 was also required as a part of B13, B14, B15). | [Q1] *I think the only thing is if we don't have a laptop for every nurse that's on, that's the big impact. There's always one in the morning that doesn't get the computer. (Interview 91)*  During busy times (e.g. morning medication rounds) there were not enough computers (laptops) on wheels (COWs) available for every nurse to use. |
|  | Properties of COWs were a barrier to taking an active eMAR to the patient when administering medication.  (Also Social/professional role and identity) | Take an active eMAR to the patient (B12) (B12 was also required as a part of B13, B14, B15). | [Q2] *If no - if they find out it’s too much equipment, too many furnishings in the room and it’s high risk for a fall for the patients, they can leave it outside and get the drawer. Just take the single drawer, put it on the COW and dispense the medication, put it back, check their MRN number and go to the patient and give it. (Interview 42)*  The COWs were bulky and when nurses judged that adding equipment (a COW) to already crowded rooms created a falls risk, they did not take the COW to the bedside to administer medication. |
|  |  |  | [Q3] *Sometimes you leave it then too because there’s no real point grabbing your clunky machine waking everyone up, as you’re dragging it down the hallway, to park it, to have the bright light shining and you confuse patients, that’s why they wake up. (Interview 39)*  The noise of the COWs, the risk of bumping into things in the dark and the brightness of the screens was likely to wake patients who were sleeping or agitated confused patients. Nurses worked around this by not taking a COW to the bedside of sleeping or confused patients. |
|  | Salient events, including patients in isolation, were barriers to taking the COW to the patient when administering medications.  (Also Social/professional role and identity) | Take an active eMAR to the patient (B12) (B12 was also required as a part of B13, B14, B15). | [Q4] *For something like common sense that’s been obvious,* ***the infection rooms we don’t take the COW. We park it outside the room*** *and we grab the medications in the kidney dish, or if we are very lucky we have another staff member that can help, she can stay with the COW and just with the medication and the second person can just take them out. (Interview 42)*  Infection control policies required equipment to be left in the isolation room or to be cleaned down when being removed from the room. This was a barrier to taking the COW into isolation rooms. |
|  | Technology characteristics e.g. short log out time at one hospital and simultaneous access to multiple users at the other hospital were barriers to signing off medication once it had been administered (once the patient was observed to consume oral medication). | Record medication administration in the eMAR following administration (B18). | [Q5] *It only lasts a while before it logs out so you can’t be taking someone to the toilet or whatever, it’ll log out and you’ve got to log back in and you’ll have lost everything if you’ve clicked anything. (Interview 03)*  At one hospital there was a short log out time. When the EMMS logged off, information entered until that point was lost and had to be re-entered. If nurses waited until after they had administered medication they risked being logged off before signing off the medication. |
|  |  |  | [Q6] *I: Another thing* ***is because with the computer everybody can access it from other terminal when you're doing something, other people probably changed something already****.* ***But with the paper chart, you are the one holding it, then no one can change an order, not unless they take it from your hand.***  *F: So if you're in the middle of a medication you could be in the middle of a medication administration using the computer and somebody in another place could change the order?*  *I: Yeah, change the order.*  *F: You were telling me that that had happened to you.*  *I: Yeah, that happened to me with the patients on anti-hypertensive drugs.*  *F: So what happened?*  *I: What happened was the doctor ceased the medication on the other terminal. I was giving out the medication and gave it to the patient, was going to sign the order and then find out the order is not there anymore. Then because the doctor on the other terminal has already ceased the order, then I need to ask the doctor to rechart another dose because it's already been given. (Interview 30)*  At one hospital more than on authorised user could be active in a patient’s eMAR at the same time. Until it has been signed off as administered in the eMAR, their colleagues do not know that the medication has been administered. Nurse 30 explains a situation in which the doctor ceased the medication order while the nurse was administering the medication (following the policy and not signing off the medication until it was administered). When the nurse went to sign off the medication the order had disappeared. To work around this problem, the nurse asked the Dr to order a STAT dose to cover the dose he/she had just administered. |
|  | Default medication administration times that did not match local context were a barrier to administering medication at the time ordered in the eMAR. Nurses either changed the medication times in the eMAR (additional steps) or administered medication and signed it off in the eMAR later when it became ‘available for administration’. | Administer medication at the prescribed time (B17).  Record medication administration in the eMAR when it has been administered (B18). | [Q7] *I: It makes it harder to - sometimes* ***with the paper charts, if the times weren’t suitable or something needed to be given with meals, we just changed the times ourselve****s or even ceased drugs if you knew they were just for 48 hours.*  *…*  *F: What about the times?*  *I:* ***Times - you can change them on the electronic chart but you think - you can only do it once after the end of the day when all the medications that you're changing have been given. So if you’ve got something TDS you can't change it at midday. You have to wait until after the last dose of the day, then you can change it for the next day****.*  *F: Whereas with the paper you could actually…*  *I: With the paper you could, yeah. It was a little bit -* ***it needs to really be given with meals or before meals or something because the electronics don’t take any account of that really****.*  *F: What do you do in that case? If you’ve got something that needs to be given but the time's wrong?*  *I:* ***I'll sometimes give it at the - what I'd think is the correct time and then sign it later and maybe change it later on****. (Interview 57)*  Specific contexts included medication times that did not match local context such as meal times. To change the medication times in the eMAR required additional steps. Nurse 57 explains that rather than changing the medication administration time in the eMAR, he/she administered the medication and signed it off later when they could sign it off as administered without having to change the time. |
|  | Time pressure and competing demands, were barriers to taking the COW to the patient for every medication administration. | Take an active eMAR to the patient (B12) (B12 was also required as a part of B13, B14, B15). | [Q8] *F: So that's one thing and you were talking about sometimes you take [the COW] with you and sometimes you don't. Okay, so what are factors that would influence which way you go?*  *I: If you're in a rush and sometimes you just can't - it's more accessible for you just to do your stuff at the computer, run to the patient and run back, for some people. (Interview 39)*  Nurses explained that when they were really busy they did not always use the EMMS as intended – this included not taking the COW to the bedside (this quote) or checking or witnessing medication administration by two nurses when required (other interview data). |
| **Social/professional role and identity** | Administering medication: Nurses without a log in were not able to access EMMS to administer medication. | Nurses endorsed to do so use the EMMS to administer medication using EMMS (B1).  The administering nurse logs into the EMMS and opens the patient’s eMAR (B2).  B2 was also required for the administering nurse as part of B14, B15, B16,B17 and B18. | [Q9] *F: That's really interesting because agency nurses can't use the electronic system, can they?*  *I: They can't. They're not happy.*  *F: So they can't give medication.*  *I: When they come here they are really, really…*  *F: Can you tell me a bit about that?*  *I: Okay. We've got the pool staff, some of them are trained for the electronic medication. They come here then they can do the medication. But the agency staff, especially someone that would be doing ICU, the high dependency one, when they come here you ask them to do the basic nursing care, they are really angry, why do I have to come here and do all the basic care, nursing care?*  *F: Really?*  *I: Yeah, because they can't do the pills. (Interview 30)*  At one hospital access to the EMMS was limited to nurses who were permanent on the units that used EMMS once they had completed the training and to select casual pool staff who frequently worked on the units that used EMMS and who had completed the training. The nurses who could not use the EMMS could not administer medications. The nurses exhibited an emotional response – they responded really angrily – because they had to do ‘basic nursing care’. At one hospital, EENs were unable to administer particular medications using EMMS (see following).  This quote is included because the described emotional response demonstrates the importance of medication administration work to nurses’ professional role and identity. |
|  | Administering medication: At one hospital enrolled nurses were blocked from signing off administration of specific medications in the eMAR. | Nurses endorsed to do so use the EMMS to administer medication using EMMS (B1)  The administering nurse logs into the EMMS and opens the patient’s eMAR (B2).  B2 was also required for the administering nurse as part of B14, B15, B16, B17 and B18. | [Q10] *I: The EENs* [endorsed enrolled nurse] *as well. There's certain things that they can and can't give. They get blocked from giving some medications. In that case, they have to come and find one of us - an RN - to log in. They can check it with us but they can't be seen as the one to administer it on [de-identified EMMS name].*  *…*  *I: … So that just go to - that affects their routine then, because they're then waiting for us to come and do something for them that might be stopping them from doing something else. So it holds them back in their patient care. Most of the EENs here will - are happy to - once they’ve had it checked by one of us, they're happy to administer it. (Interview 61)*  At one hospital endorsed enrolled nurses (EENs) were blocked from signing off specific medications in the eMAR. Nurse 61 explains that to not be able to administer medication held the nurses back from patient care and affected their routine. To work around this problem, the EENs sometimes administered medications that the registered nurses (RN) had signed off in the EMMS – the RN was recorded as having administered the medication. |
|  | Being time efficient: Being time efficient was an important part of a nurses’ professional role and identity. When using the EMMS as intended slowed nurses down it did not support professional role and identity. | Administer medication at the prescribed time (B17).  Record medication administration in the eMAR when it has been administered (B18). | [Q11] *I: I think so, I think they do crash into peer pressure a lot and they do care what other people think. I mean everyone's separate, everyone's different but I do think, especially for the juniors. I know we're renowned for eating our young and being so mean to them. I think even the juniors, they're a lot more anxious they want to get it done before anyone else, before anyone else has to check on them even though we might not even check.*  *F: So do you “check”, in inverted commas…*  *I: Yes [laughs].*  *F: So that may influence?*  *I: Yeah I think it may influence, it may be a reason why you might get things started an hour and a half early. Maybe they don't want to think they're bad nurses because they take time to read a box a little bit more than most people should. (Interview 39)*  This quote illustrates the importance of being time efficient to nurses’ professional identity. Nurses might administer medication earlier than prescribed so as not to be late administering medication. |
|  | Being time efficient: An overdue medication alert (OMA) signalled that a medication was an hour overdue. When the OMA was interpreted as a signpost to a nurse being late with a medication rather than the medication being late, nurses cut corners or delayed the medication in the eMAR to avoid or get rid of the OMA. | In as much as the visibility of the OMA led nurses to rush and take shortcuts to avoid or remove it, the OMA was a barrier to B3, B4, B8-B15, B18 as shortcuts were possible for all of these key behaviours in the medication administration process. | [Q12] *I: … Like on a busy morning shift, 9 o'clock you’re only up to two patients and there are four patients with [OMA symbols] next to it and you feel like a sense of failure maybe. In sense of like you’re slow, you’re slower than the others. Yeah like you’re no good, you've got poor time management.*  *…*  *F: So would you, does the knowledge that the [OMA symbol] is coming affect your practice, affect what you do?*  *I: Yes when they see that people try to rush, people try to rush, because it is mentally a symbol that you want to get off the computer. (Interview 31)*  The overdue medication alert (OMA) highlighted that administration of a medication was late. Nurse 31 explained that the OMA made him/her feel like a failure with poor time management. The OMA was a barrier in as much as it made some nurses rush medication administration (described above). In other interviews, nurses described using workarounds to remove the OMA from the screen – either by delaying medication administration or by cutting corners. Responses to OMAs appeared to differ between units and hospitals. |
|  | Considering individual patient preferences and needs is part of a nurses’ role and identity. Taking the COW to the bedside was considered to wake patients and increase agitation. | Take an active eMAR to the patient (B12) (B12 was also required as a part of B13, B14, B15). | [Q3] *Sometimes you leave it then too because there’s no real point grabbing your clunky machine waking everyone up, as you’re dragging it down the hallway, to park it, to have the bright light shining and you confuse patients, that’s why they wake up. (Interview 39)*  The noise of the COWs, the risk of bumping into things in the dark and the brightness of the screens was likely to wake patients who were sleeping or agitated confused patients. Nurses worked around this by not taking a COW to the bedside of sleeping or confused patients. |
|  | Considering individual patient preferences and needs is part of a nurses’ role and identity. When the EMMS was perceived to block nurses from exercising their professional judgement about appropriate timing of medication administration, it did not support their professional role and identity. | Administer medication at the prescribed time. The administering nurse records medication administration in the eMAR once it has been successfully administered (B17, B18). | [Q13] *I: Sometimes when patients want Panadol early, sometimes it won't be available in the system but you want to give it, so sometimes you just give it, then go in later and click it off. Because it is every eight, six or eight hours, but sometimes you want to give something early and you can't because it's in the system.*  *F: Because they're asking for it?*  *I: Yeah and it has to be in a set time and then sometimes you just give it and then go back in later and… (Interview 91)*  The EMMS regulated the time that medications were ‘available for administration’ and stopped them from administering medication early – even when requested to do so by the patient and within a time frame they judged to be appropriate. To work around this barrier nurses described administering medication at a time they judged to be appropriate and recorded administration at a later time when the medication became ‘available for administration’ in the system. |
|  | Promote patient safety: A nursing competency is to maintain standards of infection control. Taking the COW to the bedside (using EMMS as intended) did not align with professional role and identity when patients were isolated for infection control purposes.  (Overlap with Environmental context and resources barriers). | Take an active eMAR to the patient (B12) (B12 was also required as a part of B13, B14, B15). | [Q5] *For something like common sense that’s been obvious,* ***the infection rooms we don’t take the COW. We park it outside the room*** *and we grab the medications in the kidney dish, or if we are very lucky we have another staff member that can help, she can stay with the COW and just with the medication and the second person can just take them out. (Interview 42)*  Infection control policies required equipment to be left in the isolation room or to be cleaned down when being removed from the room. This was a barrier to taking the COW into isolation rooms. To prevent cross infection, nurses left COWs outside an isolated patient’s room. |
|  | Promote patient safety: A nursing professional competency is to provide safe care, to prevent injury by identifying, eliminating or preventing environmental hazards where possible.  (Overlap with Environmental context and resources). | Take an active eMAR to the patient (B12) (B12 was also required as a part of B13, B14, B15). | [Q2] *If no - if they find out it’s too much equipment, too many furnishings in the room and it’s high risk for a fall for the patients, they can leave it outside and get the drawer. Just take the single drawer, put it on the COW and dispense the medication, put it back, check their MRN number and go to the patient and give it. (Interview 42)*  The COWs were bulky and when nurses judged that adding equipment (a COW) to already crowded rooms created a falls risk, they did not take the COW to the bedside to administer medication. |
|  | Promote patient safety: Assessing and managing risk to deliver safe care is an important part of a nurses’ professional role. Nurses identified the potential for interruptions and subsequent risk of medication error as a barrier to taking the COW to the patient to administer medication. A nurse’s ability to manage interruptions was associated with professional experience. | Take an active eMAR to the patient (B12) (B12 was also required as a part of B13, B14, B15). | [Q14] *I suppose yeah I do. I get all the medications I can from the drug room, that's kind of the way I do it.* ***I always go to the drug room and try and get all the medications I can there, and then what I need to at the bedside, just because it saves me getting confused. But the more I'm at the bedside, patients start asking questions, and that's kind of when you lose your thoughts. So I'd rather look at the doses in the drug room where it's quiet, rather than at the bedside where other patients are*** *- can you come - and then you - I think most of the laptops have a sign on the back, saying not to interrupt. (Interview 91)*  Nurses prepared medications in the medication room or took bedside medication drawer to the COW in the corridor to avoid interruptions.  [Q15] *I: Once I'm in that patient's room* ***just because you've got a trolley there*** *as soon as you see a nurse it's like…*  *F: I've got my 10 things I need to ask you.*  *I: …I want 20 things in fact, I want the tissues here, I want the glass of water and the pillow's not - I need…*  *F: When's the doctor coming?*  *I: When's the doctor coming? I want to get out of here. I don't want to be here. These are the things that impede, you've got that. But that's going to happen. Once you see the nurse there - but that's not going to go away.* ***I'm very strict when I'm giving medications and I'm very assertive. A lot of the nurses probably can't formulate the language but that's just experience****. I'll say if I've got four ladies in a room and they're going I want this, I want that, I want that, I'll say, ‘Stop now’. I say, ‘I'm giving drugs, medications, unless I concentrate you get the wrong medication and I need to concentrate and it's not easy so I will get to you when I can but let me do this first’. (Interview 03)*  When nurses took the COW to patients’ rooms to administer medication, patients interrupted them during the medication administration process. With experience (e.g. Nurse_03), nurses were able to manage the interruptions assertively. However, inexperienced nurses had not yet formulated the language to assertively manage interruptions. |
|  | Respect for colleagues: The professional importance nurses placed on not impinging on their colleagues’ time was a barrier to asking a colleague to check medication during a busy time and, if required, to accompany the administering nurse to the bedside to check/witness administration. | Open a single patient’s eMAR at a time. With only their eMAR open prepare medication for one patient at a time immediately before intended use (includes medications requiring a second person check or witness) (B4). | [Q16] *Sometimes easier to get somebody to check - somebody else to* ***check them all beforehand rather than when it's six o'clock in the morning when it's a lot busier****. It won't be tomorrow.* ***Sometimes harder to disturb people - get somebody who's not doing something to just check it****. (Interview 57)* |
|  | Respect for colleagues: Respecting other members of the patient care team was an important part of a nurses’ professional role and identity. Nurses did not consider that logging doctors and pharmacists out of the eMAR or reclaiming the COW they had been using to administer medication (to take a COW to the bedside to administer medication) aligned with their professional identity and therefore administered medication using a desktop computer. | Take an active eMAR to the patient (B12) (B12 was also required as a part of B13, B14, B15). | [Q17] *I: Then you really want to work - that you really want to give it and then you cannot. Not like just the physical disadvantage of just moving it around but the actual medication. Only if the doctor or the pharmacist are on the same - logged in, same time as we do then whatever you give will be disappeared because they can override you. So whatever I'm doing is overridden.*  *F: So what do you do then?*  *I: Then I have to go and do it over again. I have to go all over again.*  *F: So you've got to remember to...*  *I: Yes. Whereas if the doctors and the pharmacists has nurses - like when you login and say currently being used by so and so, I just exit and I wait for another five minutes. But some doctors, if it's nurse signature, nurse login they just like ah what's this...*  *F: They just override it.*  ***I: They just override it so you lose all your work****. (Interview 45)*  Nurses explained that when colleagues logged them out of the eMAR mid task, information entered but not saved to that point had to be re-entered. Nurses did not consider that it was their role/position to log doctors and pharmacists out of the eMAR. Collegiality and team work was considered an important aspect of nursing professional behaviour. Other data highlights that rather than log a colleague out or reclaim the COW they were using, nurses will use desktop computers to administer medication. Other data demonstrates that rather than loose information when logged out, nurses signed off medication in the eMAR as administered before administering it to the patient. |
|  | Professional culture: When nurses did not consider it part of their professional role to report broken equipment, there were fewer COWs available for nurses to take to the patient when administering medication. | Take an active eMAR to the patient (B12) (B12 was also required as a part of B13, B14, B15). | [Q18] *The main maintenance of the computers is one thing. We find it difficult keeping them maintained. People a) don’t take responsibility for them so won’t initiate if they notice something wrong with it, if it is physically broken or if it is a software problem they won’t initiate it, it will just be left in the corridor to the side so it decreases the number of resources (Interview 65)*  Nurses described a professional culture in some units where reporting problems was not seen to be a collective responsibility. Rather, than report a problem with a laptop (hardware or software) so that it could be fixed, nurses left them to one side. This reduced the overall number of working COWs available for use. When there were not enough available laptops, nurses were more likely to use the desktop computers, rather than take a COW to the patient. |
|  | Professional culture that supported senior nurses or nurses with experience not taking the COW to the bedside and hindered junior nurses from saying anything | Take an active eMAR to the patient (B12) (B12 was also required as a part of B13, B14, B15). | [Q19] *There's definitely a perception, I mean I guess there's a perception where there are even paper charts when you see a senior staff, you just think they know what they're going to do, that they know what they're doing and yeah, I think that's a lot where it stems from. You think, obviously no one's perfect, so you've got to keep checking. Yeah, and you're setting, it's out of your place, if you're younger, to say, ‘Look aren't you going to take [the COW] with you?’ (Interview 39)*  Nurses described a professional culture in which it was acceptable, particularly for senior nurses, not take the COW to the patient to administer medication. The professional hierarchy in nursing made it difficult for junior nurses to ask senior nurses to follow policy, including taking a COW to the patient. |
| **Knowledge** | A lack of knowledge at one hospital about who should enter the details of the checking nurse in the eMAR was a barrier to nurses signing off medication at the time of administration. | Sign off the medication once it has been successfully administered (at the time of administration) (B18). | *But basically most times we'll check and say [de-identified] checked it, checked by [de-identified]. So I'm writing that it was checked by her. Whether she's meant to enter checked by herself I'm not really sure. I can write anybody's name down if that's the case (Interview 53)*  At one hospital there was a lack of clarity about whether the nurse who checked the medication should enter his or her own details or whether the administering nurse should complete these in the comment box. In the context of other data, this was a barrier because some nurses waited until the checking nurse had typed in their name before they signed off the medication as administered in the eMAR. |
|  | Not knowing how to use the features of the EMMS to record medication management activities was a barrier to doing so. | Recording correct status/dose of medication administered (B18). | *Number 3, if you've done something wrong, only if you know how to correct it, time to correct properly, because it does take time to go back and correct it and if the patient didn't take the medication then you have to go back and do all that, I found some, most of my colleagues, when they have to go and do that, they complain a lot, they say oh –they become negative about it. Often times they come to me, especially agency staff if they've got an electronic medication chart password, and they don't know how to do it. When they come to me, I've been approached a couple of times to show them how to do it. So that also generates some workloads. (Interview 31)*  Some nurses did not know how to correct incorrect entries that they have made in the eMAR. For example, when the patient refused a medication, some nurses did not know how to correct an erroneous entry. |
|  | Unfamiliarity with information technology (IT) generally posed a barrier to using EMMS at all steps of the medication administration process. | Use the EMMS to administer medication – a lack of knowledge about using IT would pose a potential barrier to all steps of the medication administration process (B1-B18). | *Not from a personal point of view but from a general, I can see it would be difficult with a generational change. This day and age, it dominates our life, just looking at the phones and how much they've changed over the last 24, 36 months and the iPhones. It's been life-changing. But if people are at an older age or a different generation where they're not used as much, it could be a boundary. (Interview 21)*  A lack of knowledge about how to use IT generally was a barrier to using EMMS. It was believed that older nurses would find it more difficult to use EMMS because they are not as familiar with technology as younger nurses. |
|  | Limited knowledge of the meaning of some of the icons was a barrier to nurses recording and communicating information about medication at one hospital. | Check medication preparation and administration icons in the eMAR and record and communicate relevant information about medication administration (B8 and B18). | *F: There's also sometimes I see a little yellow triangle on the - you know there's an order, like there'll be that blue box with you know I don't know 1000 milligrams on but sometimes there's a little yellow triangle in it.*  *I: I'm not sure.*  *F: I'll have to have a look.*  *I: Does it look like any of them? [Shows an ID sized laminated card displaying symbols and corresponding meaning]*  *F: Nope.*  *I: I'm not sure then.*  *F: MAR note. I have seen that one.*  *I: Yeah they've just made a note or it's got a pin attached so basically I don't know whether that's from us or not. I never pay much attention to those things. (Interview 53)*  Nurse 53 is unsure of the meaning of some of the icons and symbols in the medication order. |
| **Beliefs about consequences** | A barrier to taking the COW to the patient to administer medication is a belief that there will not be negative consequences of not doing. Conversely with some medications the belief that not taking the COW to the patient and co-checking the medication will have negative consequences. | Take an active eMAR to the patient (B12) (B12 was also required as a part of B13, B14, B15). | *F: So would there be some times when you would go – in these circumstances I always take the laptop but in these other circumstances I don’t?*  *I: I always take it for chemo - that is about it …*  *F: Now why is that?*  *I: Because chemotherapy is – I don’t know, I guess it is just more dangerous, it is a more dangerous sort of drug so you need to be checking, to check with two people at the bedside.*  Whether or not the nurse takes the COW to the patient to administer medication is influenced by the believed consequences of not doing so. Not doing so with chemotherapy is believed to have potentially more dangerous consequences. |
|  | A barrier to taking the COW to the patient to administer medication was a belief that using the COW for every medication administration would have negative consequences (e.g. medications would be administered late; patients would not be happy and would not receive adequate care). | Take an active eMAR to the patient (B12) (B12 was also required as a part of B13, B14, B15). | *There’s no excuse* [for not taking the COW to the patient]. *I mean, you can’t say - it’s not a matter that you know that patient and you’ve been with the patient for three or four months but you have to take it* [the COW to the patient]*. You tell me, if I - like for instance, from A to Z, alright. I’ve been looking after one, two and then from that patient to this patient. Now, if I start it this way* [take the COW to the patient] *would you reckon that I will finish my medication by the time? (Interview 33)*  The nurse explains that if he/she took the COW to the patient every time they administered medication to them, they would not be able to administer all of their patients’ medications on time. In this quote, it is evident that the nurse knows what should be done but believes that the consequences of doing so would be negative (the medications will be late). |
|  |  |  | *But if you're too absorbed and you're struggling with it and you find that you didn't have enough time to look after your patient, so the quality of care will be affected. So when the patient sees you just focusing on the computer, eyes fixed on computer, talking to them eyes fixed on the computer, some patients might take it as a negative thing. So it's two-way. Safety I think it's safer, more safer than not. (Interview 31)*  There is a belief that a consequence of using the EMMS is that patient care can be neglected and that patients have a negative perception of nurses using computers - that the nurse is focused on the computer rather than patient care. |
|  | Nurses identified the potential for interruptions and subsequent risk of medication error as a barrier to taking the COW to the patient to administer medication.  (Overlap with Social/professional role and identity). | Take an active eMAR to the patient (B12) (B12 was also required as a part of B13, B14, B15). | *I suppose yeah I do. I get all the medications I can from the drug room, that's kind of the way I do it.* ***I always go to the drug room and try and get all the medications I can there, and then what I need to at the bedside, just because it saves me getting confused. But the more I'm at the bedside, patients start asking questions, and that's kind of when you lose your thoughts. So I'd rather look at the doses in the drug room where it's quiet, rather than at the bedside where other patients are*** *- can you come - and then you - I think most of the laptops have a sign on the back, saying not to interrupt. (Interview 91)*  Nurses prepared medications in the medication room or took bedside medication drawer to the COW in the corridor to avoid interruptions. |
|  | A barrier to EMMS implementation was a belief that a consequence of implementation of the EMMS was that nurses spent more time looking at the EMMS than looking after patients. | Implementation of EMMS | *I: Yeah, I just think that people tend to use the COW more, stay in front of the COW more looking up things nowadays. Then sometimes they just ignore the bedside care. Sometimes people ring their buzzer and they don't go and answer. (Interview 30)* |
| **Beliefs about capabilities** | Nurses’ confidence in their professional experience and medication knowledge and capabilities to administer medication safely without taking the COW to the bedside was a barrier to taking the COW to the patient to administer medication. | Take an active eMAR to the patient (B12) (B12 was also required as a part of B13, B14, B15). | *I: When there are certain instructions - if you do drug administration and your knowledge of drugs is poor, it would be a good idea to take it* [the COW] *with you, because it does have warnings on it; and how you should do things. But once you do things a number of times, you do learn to remember it. Like, the right doses - 100 millilitres over an hour or two hours, depending on what the drug is; if you have got to mix it up with saline and not water and those sorts of things.* ***With experience you get to know it. It's when your knowledge is not great; if you are fairly new to the whole thing.***  *F: Then it is not okay to work around the system?*  *I: Yeah. Until you have more knowledge. It is something that is valid, isn't it, knowledge. You can't measure it, but it is there. I do know a lot of things about drugs, but new nurses don't. (Interview 24)*  Taking the COW to the patient when administering medication was believed to be less necessary for nurses with professional experience and a sound knowledge about medication. |
|  | Lack of confidence in their ability to request that a colleague follow policy was a barrier to accompanying a colleague to the patient to check/witness administration of medications when required. As a result nurses signed off medication in the eMAR before it was administered. | Accompany the administering nurse to the patient to check/witness administration of medication (B14, B15). | *But some people are really assertive you know, “Just wait a moment I'll come with you". And when they say that I always, you know, have a certain secret admiration. I wish that I could do that. (Interview 31)*  Nurse 31 does not believe he/she has the capability to assertively ask a colleague to wait while he/she accompanies him/her to the patient to check/witness medication administration. |
| **Social influences** | Social influences including pressure, modelling, comparison, social norms influenced whether or not nurses took the COW to the patient, witnessed or checked medication administration or signed off medication after observed administration. | Accompany the administering nurse to the patient (with the COW) to check/witness administration of medication (B14, B15). | *Another thing is, also* ***depending on who you are working with****, some of them are really pedantic, let's take* ***everything*** [including computer] *to the patient. Some of them, even if you want them to come with you, you've basically got to lasso them to drag them, so it's just easier for you to check it on their computer, or at yours and then run away. (Interview 39)*  Nurses’ attitudes and willingness to follow policies that required them to take the COW together to the patient for administration of medications requiring a witness influenced whether or not their colleagues did so. |
|  |  | Sign off the medication once it has been successfully administered (at the time of administration) B18. | *I: Then you really want to work - that you really want to give it and then you cannot. Not like just the physical disadvantage of just moving it around but the actual medication. Only if the doctor or the pharmacist are on the same - logged in, same time as we do then whatever you give will be disappeared because they can override you. So whatever I'm doing is overridden.*  *F: So what do you do then?*  *I: Then I have to go and do it over again. I have to go all over again.*  *F: So you've got to remember to...*  *I: Yes. Whereas if the doctors and the pharmacists has nurses - like when you login and say currently being used by so and so, I just exit and I wait for another five minutes. But some doctors, if it's nurse signature, nurse login they just like ah what's this...*  *F: They just override it.*  ***I: They just override it so you lose all your work****. (Interview 45)*  Nurses explained that when colleagues logged them out of the eMAR mid task, information entered but not saved to that point had to be re-entered.  Other data demonstrates that rather than loose information when logged out, nurses signed off medication in the eMAR as administered before administering it to the patient. |
| **Memory, attention and decision processes** | Nurses forget to follow policy when administering medication. | Take an active eMAR to the patient (B12) (B12 was also required as a part of B13). | *As I said to you, a lot of people they - for instance, I did the medication. In the morning everywhere I did go I check each and every thing. Well, it depends on what you do. But to be honest, sometimes to me I sometimes forget to do the proper way. I just give the medication and then I realise later what did you do? You should have done it the proper way. You should have done it this way. So it’s human nature. You do make mistakes. (Interview 33)*  The nurse forgets to follow policy when using the EMMS [in context, the nurse is referring to taking the COW to the patient to check the 5Rs and allergies]. |
|  | Nurses are not aware or forget that a STAT medication has been ordered. There is no alert to remind them that it has not been administered. This is a barrier to them administering it at the prescribed time (STAT). | Administer medications according to a timeframe prescribed in the eMAR (B17). | *I: …* ***At times, I find possibly STAT medication can get misplaced*** *- not the actual medication but the order.*  *F: And that's different from when it was on paper?*  *I: Yeah. It is different because nine times out of 10, if a doctor had written a STAT medication, they would tell you and they would actually physically hand the med chart to you. Now they may say look, the STAT medication has being charted for Bed 9 and then they'll just physically walk away. You go yeah, no worries, but* ***you've got nine or 10 other things on your mind and that's how it can be overlooked****. (Interview 21)*  The paper chart acted as a physical prompt to remind nurses that a STAT medication had been ordered. The EMMS does not have an alert to remind nurses to administer STAT medication and they forget. |
| **Emotion** | Being ‘sick of it’ and burnout (emotions) were barriers to nurses following the requirement for two nurses to attend the patient with the COW to check/witness medication administration for relevant medications. | Accompany the administering nurse to the patient (with the COW) to check/witness administration of medication (B14, B15). | *So you are more likely to stick to the rule* [to witness/check medication administration] *whereas with the senior staff, because the senior staff, people who are more senior than me, some people they’re so sick of it, they're burnt out, they are so busy, and they just want to, make sure the patient is being–they are really good nurses. (Interview 31)*  When nurses were sick of it or burnt out, they were less likely to follow the requirement that two nurses go to the patient with the COW to check/witness medication administration for medications to which this was applicable. |
|  | The overdue medication alert elicited a negative emotional response, which was a barrier to nurses using the OMA as a reminder, instead rushing or delaying medications to remove it. | In as much as avoiding the negative emotion led nurses to rush and take shortcuts to avoid or remove it, the emotion was a barrier to B3, B4, B8-B15, B18-  B19 as shortcuts were possible for all of these key behaviours in the medication administration process. | *I: The [OMA symbol]? I don’t know. They're just irritating when you haven’t had a chance to do them and it comes. All of a sudden it says, you haven’t given these drugs. You're naughty.*  *F: They don’t bother you at all then?*  *I: Just mildly irritating when you’ve just been busy doing other things and you know you’ve got drugs to do but you haven’t got around to it just in time or the patient's been away. You feel like yelling, ‘I know, I know, I know. Shut up’. (Interview 57)*  The OMA produced an emotional response because it reminded nurses that they had not given medication on time. Negative emotion causes nurses avoid the OMA, which has been described in other quotes (e.g. Nurse 31). |
| **Intention** | The nurse did not intend to use the eMAR when administering medication – there was no intention to take an active eMAR to the patient, check the information about the medication or enter the medication as administered at time of administration. | Take an active eMAR to the patient (B12) (B12 was also required as a part of B13, B14, B15).  Check medication preparation and administration instructions and alerts/icons in the eMAR and check the purpose, action and safe dose range of the medication to be administered prior to administering the medication (B9, B10). | *I know one person who does - prints off pretty well every time* [he/she] *gives drugs.* [He/she] *doesn’t like the computer so* [he/she] *does it - prints them off and does it from the paper. (Interview 57)*  Rather than take the COW to the patient when administering medication and signing off the medication in the eMAR once it was administered, the nurse described in this quote printed off the medication order and administered medication from the print out and reconciled the eMAR later in the shift. That the nurse does this “pretty well every time” suggests an intention not to use the active eMAR when administering medication. |
