# Supplementary material for: Applying the Theoretical Domains Framework to identify barriers and targeted interventions to enhance nurses’ use of electronic medication management systems in two Australian hospitals
Source: Implement Sci. 2017 Mar 27;12:42. doi: 10.1186/s13012-017-0572-1 (PMC5368903; doi:10.1186/s13012-017-0572-1)
Supplement: Supplementary file 2 — Proposed interventions (modes of delivery of BCTs) mapped to barriers to EMMS use. Two domains of the TDF emerged more strongly than the others in capturing barriers to nurses’ use of EMMS: Environmental Context and Resources, and Social/Professional Role and Identity. In-text references to specific examples (Additional file 2: Table S7) are given in the form TableNumber_QuoteNumber (e.g. T7_Q1)]. We then identify BCTs that have been shown to be effective in influencing these two theoretical domains. For each barrier, we present an example of a BCT operationalised into a potential context-specific intervention strategy. To distinguish them from the specific targeted interventions designed to deliver them, BCTs are italicised in brackets. Additional examples are provided in (Additional file 2: Table S7). (DOCX 56 kb) [file 13012_2017_572_MOESM2_ESM.docx]

| **Additional file 2: Table S7. Proposed interventions (modes of delivery of BCTs) mapped to barriers to EMMS use** | | | | | |
| --- | --- | --- | --- | --- | --- |
| **Barrier label** | **Barrier Description** | **Barrier to corresponding behaviour description and ID (Table 5)** | **[Quote in text reference] *Example quote***  **Description** | **Examples of technique label and definition Michie et al (2013) [43]**  **(BCT mapped to TDF domain: $Michie et al (2008) [37]; #Yorkshire Quality and Safety Research Group (2013)[49]; @Cane et al (2015)[42])**  ***Where relevant additional notes to explain choice of BCT*** | **Example of potential interventions (modes of delivery of BCT) to overcome barriers**  **(Where applicable: references for examples of studies that have used the mode of delivery of BCT)** |
| **Environmental context and resources** | Unavailability of COWs was a barrier to taking an active eMAR to the patient when administering medication. | Take an active eMAR to the patient (B12) (B12 was also required as a part of B13, B14, B15). | [Q1] *I think the only thing is if we don't have a laptop for every nurse that's on, that's the big impact. There's always one in the morning that doesn't get the computer. (Interview 91)*  During busy times (e.g. morning medication rounds) there were not enough computers (laptops) on wheels (COWs) available for every nurse to use. | **Adding objects to the environment ($; @)**  “Add objects to the environment in order to facilitate performance of the behaviour” [43:S17].  *Cane et al 2015 assign “Antecedents” to Environmental Context and Resources. This is an example of a BCT in the Antecedent BCT grouping in the Michie et al (2013) taxonomy* | Provide additional mobile electronic devices.  (A study by Taylor et al (2013) operationalized tis BCT by adding litmus strips to test Ph for nasogastric tube placement [45]). |
|  | Properties of COWs was a barrier to taking an active eMAR to the patient when administering medication.  (Also Social/professional role and identity) | Take an active eMAR to the patient (B12) (B12 was also required as a part of B13, B14, B15). | [Q2] *If no - if they find out it’s too much equipment, too many furnishings in the room and it’s high risk for a fall for the patients, they can leave it outside and get the drawer. Just take the single drawer, put it on the COW and dispense the medication, put it back, check their MRN number and go to the patient and give it. (Interview 42)*  The COWs were bulky and when nurses judged that adding equipment (a COW) to already crowded rooms created a falls risk, they did not take the COW to the bedside to administer medication. | **Restructuring the physical environment ($; @)**  “Change, or advise to change the physical environment in order to facilitate performance of the wanted behavior or create barriers to the unwanted behavior (other than prompts/cues, rewards and punishments)” [43:S16]. | Provide smaller mobile electronic devices such as tablets. |
|  |  |  | [Q3] *Sometimes you leave it then too because there’s no real point grabbing your clunky machine waking everyone up, as you’re dragging it down the hallway, to park it, to have the bright light shining and you confuse patients, that’s why they wake up. (Interview 39)*  The noise of the COWs, the risk of bumping into things in the dark and the brightness of the screens was likely to wake patients who were sleeping or agitated confused patients. Nurses worked around this by not taking a COW to the bedside of sleeping or confused patients. |  |  |
|  | Salient events, including patients in isolation, were barriers to taking the COW to the patient when administering medications.  (Also Social/professional role and identity) | Take an active eMAR to the patient (B12) (B12 was also required as a part of B13, B14, B15). | [Q4] *For something like common sense that’s been obvious,* ***the infection rooms we don’t take the COW. We park it outside the room*** *and we grab the medications in the kidney dish, or if we are very lucky we have another staff member that can help, she can stay with the COW and just with the medication and the second person can just take them out. (Interview 42)*  Infection control policies required equipment to be left in the isolation room or to be cleaned down when being removed from the room. This was a barrier to taking the COW into isolation rooms. | **Adding objects to the environment ($; @)**  “Add objects to the environment in order to facilitate performance of the behaviour” [43:S17].  **Restructuring the physical environment ($; @)**  “Change, or advise to change the physical environment in order to facilitate performance of the wanted behavior or create barriers to the unwanted behavior (other than prompts/cues, rewards and punishments)” [43:S16]. | Provide more mobile electronic devices so that there are enough for isolation rooms to have a dedicated mobile device.  Provide smaller mobile electronic devices such as tablets that can be encased in disposable covers. |
|  | Technology characteristics e.g. short log out time (at one hospital) and simultaneous access to multiple users (at the other hospital) were barriers to signing off medication once it had been administered (once the patient was observed to consume oral medication). | Record medication administration in the eMAR following administration (B18). | [Q5] *It only lasts a while before it logs out so you can’t be taking someone to the toilet or whatever, it’ll log out and you’ve got to log back in and you’ll have lost everything if you’ve clicked anything. (Interview 03)*  At one hospital there was a short log out time. When the EMMS logged off, information entered until that point was lost and had to be re-entered. If nurses waited until after they had administered medication they risked being logged off before signing off the medication. | **Restructuring the physical environment ($; @)**  “Change, or advise to change the physical environment in order to facilitate performance of the wanted behavior or create barriers to the unwanted behavior (other than prompts/cues, rewards and punishments)” [43:S16]. | Lengthen the log out time or introduce an automatic screen lock without log out. |
|  |  |  | [Q6] *I: Another thing* ***is because with the computer everybody can access it from other terminal when you're doing something, other people probably changed something already****.* ***But with the paper chart, you are the one holding it, then no one can change an order, not unless they take it from your hand.***  *F: So if you're in the middle of a medication you could be in the middle of a medication administration using the computer and somebody in another place could change the order?*  *I: Yeah, change the order.*  *F: You were telling me that that had happened to you.*  *I: Yeah, that happened to me with the patients on anti-hypertensive drugs.*  *F: So what happened?*  *I: What happened was the doctor ceased the medication on the other terminal. I was giving out the medication and gave it to the patient, was going to sign the order and then find out the order is not there anymore. Then because the doctor on the other terminal has already ceased the order, then I need to ask the doctor to rechart another dose because it's already been given. (Interview 30)*  At one hospital more than on authorised user could be active in a patient’s eMAR at the same time. Until it has been signed off as administered in the eMAR, their colleagues do not know that the medication has been administered. Nurse 30 explains a situation in which the doctor ceased the medication order while the nurse was administering the medication (following the policy and not signing off the medication until it was administered). When the nurse went to sign off the medication the order had disappeared. To work around this problem, the nurse asked the Dr to order a STAT dose to cover the dose he/she had just administered. | **Restructuring the physical environment ($; @)**  “Change, or advise to change the physical environment in order to facilitate performance of the wanted behavior or create barriers to the unwanted behavior (other than prompts/cues, rewards and punishments)” [43:S16]. | Restructure the EMMS to allow one authorised user to be active in a patient’s eMAR at a time. Consider giving additional authorised users ‘read only’ access when another use is actively using a patient’s eMAR. |
|  | Default medication administration times that did not match local context were a barrier to administering medication at the time ordered in the eMAR. Nurses either changed the medication times in the eMAR (additional steps) or administered medication and signed it off in the eMAR later when it became ‘available for administration’. | Administer medication at the prescribed time (B17).  Record medication administration in the eMAR when it has been administered (B18). | [Q7] *I: It makes it harder to - sometimes* ***with the paper charts, if the times weren’t suitable or something needed to be given with meals, we just changed the times ourselve****s or even ceased drugs if you knew they were just for 48 hours.*  *…*  *F: What about the times?*  *I:* ***Times - you can change them on the electronic chart but you think - you can only do it once after the end of the day when all the medications that you're changing have been given. So if you’ve got something TDS you can't change it at midday. You have to wait until after the last dose of the day, then you can change it for the next day****.*  *F: Whereas with the paper you could actually…*  *I: With the paper you could, yeah. It was a little bit -* ***it needs to really be given with meals or before meals or something because the electronics don’t take any account of that really****.*  *F: What do you do in that case? If you’ve got something that needs to be given but the time's wrong?*  *I:* ***I'll sometimes give it at the - what I'd think is the correct time and then sign it later and maybe change it later on****. (Interview 57)*  Specific contexts included medication times that did not match local context such as meal times. To change the medication times in the eMAR required additional steps. Nurse 57 explains that rather than changing the medication administration time in the eMAR, he/she administered the medication and signed it off later when they could sign it off as administered without having to change the time. | **Prompts/cues (#; @)**  “Introduce or define environmental or social stimulus with the purpose of prompting or cueing the behavior. The prompt or cue would normally occur at the time or place of performance”[43:S9]. | Include an alert in the EMMS when the timing of medication administration is influenced by context (e.g. meal times) to remind the doctors to take into account local context when prescribing medication.  Provide stickers on computers to remind doctors to consider local context when prescribing (e.g. “06:30 is one hour before breakfast on Unit X”). [62] |
|  |  |  |  |  |  |
|  | Time pressure and competing demands were barriers to taking the COW to the patient for every medication administration. | Take an active eMAR to the patient (B12) (B12 was also required as a part of B13, B14, B15). | [Q8] *F: So that's one thing and you were talking about sometimes you take [the COW] with you and sometimes you don't. Okay, so what are factors that would influence which way you go?*  *I: If you're in a rush and sometimes you just can't - it's more accessible for you just to do your stuff at the computer, run to the patient and run back, for some people. (Interview 39)*  Nurses explained that when they were really busy they did not always use the EMMS as intended – this included not taking the COW to the bedside (this quote) or checking or witnessing medication administration by two nurses when required (other interview data). | **Adding objects to the environment ($; @)**  “Add objects to the environment in order to facilitate performance of the behaviour”[43:S17].  **Prompts/cues (#; @)**  “Introduce or define environmental or social stimulus with the purpose of prompting or cueing the behavior. The prompt or cue would normally occur at the time or place of performance” [43:S9]. | Streamline processes to facilitate medication administration tasks by ensuring there are resources and staff available at medication administration times e.g. appointing one nurse to have the role of checking/witnessing medications. *This is adding a person to medication administration process (not necessarily a new person – although that might be ideal) – i.e. making them available for checking during medication rounds.[45]*  The buddied nurses would prompt each other to follow witnessing and checking procedure.  *The buddied nurses would prompt each other to follow witnessing and checking procedure.* |
| **Social/professional role and identity** | **Administering medication:** Nurses without a log in were not able to access EMMS to administer medication. | Nurses endorsed to do so use the EMMS to administer medication using EMMS (B1).  The administering nurse logs into the EMMS and opens the patient’s eMAR (B2).  B2 was also required for the administering nurse as part of B14, B15, B16,B17 and B18. | [Q9] *F: That's really interesting because agency nurses can't use the electronic system, can they?*  *I: They can't. They're not happy.*  *F: So they can't give medication.*  *I: When they come here they are really, really…*  *F: Can you tell me a bit about that?*  *I: Okay. We've got the pool staff, some of them are trained for the electronic medication. They come here then they can do the medication. But the agency staff, especially someone that would be doing ICU, the high dependency one, when they come here you ask them to do the basic nursing care, they are really angry, why do I have to come here and do all the basic care, nursing care?*  *F: Really?*  *I: Yeah, because they can't do the pills. (Interview 30)*  At one hospital access to the EMMS was limited to nurses who were permanent on the units that used EMMS once they had completed the training and to select casual pool staff who frequently worked on the units that used EMMS and who had completed the training. The nurses who could not use the EMMS could not administer medications. The nurses exhibited an emotional response – they responded really angrily – because they had to do ‘basic nursing care’. At one hospital, EENs were unable to administer particular medications using EMMS (see following).  This quote is included because the described emotional response demonstrates the importance of medication administration work to nurses’ professional role and identity. | **Information about other’s approval (#)**  Provide information about others’ approval – information about what people think (like, approval or disapproval) about the person’s behaviour.^%^ | Provide information on what others (e.g. patients, opinion leaders) think about the importance of all nursing care – including non-medication related care – on patients’ experiences of hospitalisation. For example, collect and provide feedback on patient reports of positive experiences of holistic care (including basic nursing care).  Demonstrate opinion leaders’ esteem for delivery of non-medication care through presentation of awards to nurses who perform non-medication related activities well (e.g. nurse voted the best at giving good pressure area care). Opinion leaders, patients, or managers should present awards to provide information about other’s approval for doing ‘basic nursing care’ well and its importance as a professional characteristic of a good nurse. |
|  |  |  |  | **BCT Grouping = Identity(*)**  **BCT = Incompatible beliefs**  “Draw attention to discrepancies between current or past behavior and self-image, in order to create discomfort (includes ‘Cognitive dissonance’)” [1:S17].^ | Draw attention to a discrepancy between expressed beliefs that nursing is about holistic patient care and emotive reactions that suggest that medication administration is pivotal to being a good nurse. This could be achieved during professional development sessions to ask nurses to write down activities of a good nurse and to compare the emotional response they feel if blocked from doing these e.g. mouth care and pressure area care with not being able to administer medications. |
|  | **Administering medication:** At one hospital enrolled nurses were blocked from signing off administration of specific medications in the eMAR. | Nurses endorsed to do so use the EMMS to administer medication using EMMS (B1)  The administering nurse logs into the EMMS and opens the patient’s eMAR (B2).  B2 was also required for the administering nurse as part of B14, B15, B16, B17 and B18. | [Q10] *I: The EENs* [endorsed enrolled nurse] *as well. There's certain things that they can and can't give. They get blocked from giving some medications. In that case, they have to come and find one of us - an RN - to log in. They can check it with us but they can't be seen as the one to administer it on [de-identified EMMS name].*  *…*  *I: … So that just go to - that affects their routine then, because they're then waiting for us to come and do something for them that might be stopping them from doing something else. So it holds them back in their patient care. Most of the EENs here will - are happy to - once they’ve had it checked by one of us, they're happy to administer it. (Interview 61)*  At one hospital endorsed enrolled nurses (EENs) were blocked from signing off specific medications in the eMAR. Nurse 61 explains that to not be able to administer medication held the nurses back from patient care and affected their routine. To work around this problem, the EENs sometimes administered medications that the registered nurses (RN) had signed off in the EMMS – the RN was recorded as having administered the medication. | **Information about other’s approval (#)**  “Provide information about what other people think about the behavior. The information clarifies whether others will like, approve or disapprove of what the person is doing or will do“ [43:S8].^%^ | Use visual images to convey disapproval of nursing professional bodies, lawyers and managers of nurses signing off medication as administered in the EMMS when a colleague actually administered the medication. Posters with images of opinion leaders offering the mottos for example: *“Say ‘NO’ to signing off medication unless you administered it.”*/ *“Sign it only if you administered it”*. Position posters where nurses prepare medications requiring another nurse to check or witness (e.g. in the medication room) where nurses tend to prepare the medications that cannot be administered by ENs. |
|  |  |  |  | **Identification of self as role model (#)**  “Inform that one's own behavior may be an example to others” [43:S17]. | Inform senior nurses that their medication administration behaviour is a role model for others – encourage them to identify the importance of their actions in shaping their unit’s professional culture. Invite senior nurses to present information about medication administration as a part of ongoing education to encourage them to identify as role models. |
|  |  |  |  | **Social support (practical) ($, #)**  “Advise on, arrange, or provide **practical** help *(e.g. from friends, relatives, colleagues, ‘buddies’ or staff)* for performance of the behaviour” [43:S6]. | Suggest ways in which an EEN and RN can team up to share medication administration for their allocated patients during a shift. The RN could then administer medications that their partner is not permitted to administer. |
|  | **Being time efficient:** Being time efficient was an important part of a nurses’ professional role and identity. When using the EMMS as intended slowed nurses down it did not support professional role and identity. | Administer medication at the prescribed time (B17).  Record medication administration in the eMAR when it has been administered (B18). | [Q11] *I: I think so, I think they do crash into peer pressure a lot and they do care what other people think. I mean everyone's separate, everyone's different but I do think, especially for the juniors. I know we're renowned for eating our young and being so mean to them. I think even the juniors, they're a lot more anxious they want to get it done before anyone else, before anyone else has to check on them even though we might not even check.*  *F: So do you “check”, in inverted commas…*  *I: Yes [laughs].*  *F: So that may influence?*  *I: Yeah I think it may influence, it may be a reason why you might get things started an hour and a half early. Maybe they don't want to think they're bad nurses because they take time to read a box a little bit more than most people should. (Interview 39)*  This quote illustrates the importance of being time efficient to nurses’ professional identity. Nurses might administer medication earlier than prescribed so as not to be late administering medication. | **BCT Grouping = Identity***  **BCT = Incompatible beliefs**  “Draw attention to discrepancies between current or past behavior and self-image, in order to create discomfort (includes ‘Cognitive dissonance’)”[43:S17]. | Use persuasive communication during professional development sessions to draw attention to the discrepancies between rushing and administering medication earlier than prescribed and self-image as a nurse who takes the time to administer medication safely. |
|  | **Being time efficient:** An overdue medication alert (OMA) signalled that a medication was an hour overdue. When the OMA was interpreted as a signpost to a nurse being late with a medication rather than the medication being late, nurses cut corners or delayed the medication in the eMAR to avoid or get rid of the OMA. | In as much as the visibility of the OMA led nurses to rush and take shortcuts to avoid or remove it, the OMA was a barrier to B3, B4, B8-B15, B18 as shortcuts were possible for all of these key behaviours in the medication administration process. | [Q12] *I: … Like on a busy morning shift, 9 o'clock you’re only up to two patients and there are four patients with [OMA symbols] next to it and you feel like a sense of failure maybe. In sense of like you’re slow, you’re slower than the others. Yeah like you’re no good, you've got poor time management.*  *…*  *F: So would you, does the knowledge that the [OMA symbol] is coming affect your practice, affect what you do?*  *I: Yes when they see that people try to rush, people try to rush, because it is mentally a symbol that you want to get off the computer. (Interview 31)*  The overdue medication alert (OMA) highlighted that administration of a medication was late. Nurse 31 explained that the OMA made him/her feel like a failure with poor time management. The OMA was a barrier in as much as it made some nurses rush medication administration (described above). In other interviews, nurses described using workarounds to remove the OMA from the screen – either by delaying medication administration or by cutting corners. Responses to OMAs appeared to differ between units and hospitals. | **BCT Grouping = Identity***  **BCT = Framing/reframing**  Suggest the deliberate adoption of a perspective or new perspective on behavior (e.g. its purpose) in order to change cognitions or emotions about performing the behavior (includes ‘Cognitive structuring’)” [43:S17].^ | Prompt nurses to deliberately adopt a new perspective on the interpretation of the overdue medication alert (OMA). Convey this message during professional development sessions emphasising the OMA as a reminder that medication is late rather than a signpost that the nurse is late. |
|  |  |  |  | **BCT Grouping = Identity***  **BCT = Incompatible beliefs**  “Draw attention to discrepancies between current or past behavior and self-image, in order to create discomfort (includes ‘Cognitive dissonance’)” [43:S17].^ | Use persuasive communication during professional development sessions to draw attention to the discrepancies between rushing to get rid of the OMA and self-image as a nurse who takes the time to administer medication safely. |
|  |  |  |  | **Social comparison (#;$)**  “Draw attention to others’ performance to allow comparison with the person’s own performance” [43:S8]. | Provide opportunities for social comparison through a facilitated workshop [63]. Draw attention to others’ attitudes to the OMA as a reminder that medication has yet to be administered rather than signposting an individual nurse as late with medications.  Hold a between unit competition to design a poster or screen saver depicting OMA as a reminder alert rather than signpost to tardy nurses. |
|  | **Considering individual patient preferences and needs** is part of a nurses’ role and identity. Taking the COW to the bedside was considered to wake patients and increase agitation. | Take an active eMAR to the patient (B12) (B12 was also required as a part of B13, B14, B15). | [Q3] *Sometimes you leave it then too because there’s no real point grabbing your clunky machine waking everyone up, as you’re dragging it down the hallway, to park it, to have the bright light shining and you confuse patients, that’s why they wake up. (Interview 39)*  The noise of the COWs, the risk of bumping into things in the dark and the brightness of the screens was likely to wake patients who were sleeping or agitated confused patients. Nurses worked around this by not taking a COW to the bedside of sleeping or confused patients. | **BCT Grouping = Identity (*)**  **BCT = Incompatible beliefs**  “Draw attention to discrepancies between current or past behavior and self-image, in order to create discomfort (includes ‘Cognitive dissonance’)” [43:17].^ | Use persuasive communication to draw attention to the discrepancies between the potential implications of not taking the COW to the bedside to check the 5Rs (potential medication error) and the importance of safe medication administration to self-image of a professional nurse. The mode of delivery may include a DVD or live presentation during facilitated professional development sessions. |
|  | **Considering individual patient preferences and needs** is part of a nurses’ role and identity. When the EMMS was perceived to block nurses from exercising their professional judgement about appropriate timing of medication administration, it did not support their professional role and identity. | Administer medication at the prescribed time. The administering nurse records medication administration in the eMAR once it has been successfully administered (B17, B18). | [Q13] *I: Sometimes when patients want Panadol early, sometimes it won't be available in the system but you want to give it, so sometimes you just give it, then go in later and click it off. Because it is every eight, six or eight hours, but sometimes you want to give something early and you can't because it's in the system.*  *F: Because they're asking for it?*  *I: Yeah and it has to be in a set time and then sometimes you just give it and then go back in later and… (Interview 91)*  The EMMS regulated the time that medications were ‘available for administration’ and stopped them from administering medication early – even when requested to do so by the patient and within a time frame they judged to be appropriate. To work around this barrier nurses described administering medication at a time they judged to be appropriate and recorded administration at a later time when the medication became ‘available for administration’ in the system. | **BCT Grouping = Identity**  **BCT = Framing/reframing (*)**  “Suggest the deliberate adoption of a perspective or new perspective on behavior (e.g. its purpose) in order to change cognitions or emotions about performing the behavior (includes ‘Cognitive structuring’)” [43:S3].^ | Emphasise that the EMMS is in place to protect patients and staff – if the record is not accurate its ability to do so is limited. During professional development sessions present a DVD featuring a lawyer presenting a persuasive message about the importance of the EMMS in protecting the patients from medication error and the staff from potential legal retribution through accurate recording of medication administration. [63] |
|  | **Promote patient safety:** A nursing competency is to maintain standards of infection control. Taking the COW to the bedside (using EMMS as intended) did not align with professional role and identity when patients were isolated for infection control purposes.  (Overlap with Environmental context and resources barriers). | Take an active eMAR to the patient (B12) (B12 was also required as a part of B13, B14, B15). | [Q5] *For something like common sense that’s been obvious,* ***the infection rooms we don’t take the COW. We park it outside the room*** *and we grab the medications in the kidney dish, or if we are very lucky we have another staff member that can help, she can stay with the COW and just with the medication and the second person can just take them out. (Interview 42)*  Infection control policies required equipment to be left in the isolation room or to be cleaned down when being removed from the room. This was a barrier to taking the COW into isolation rooms. To prevent cross infection, nurses left COWs outside an isolated patient’s room. | **BCT Grouping = Identity**  **BCT = Framing/reframing (*)**  “Suggest the deliberate adoption of a perspective or new perspective on behavior (e.g. its purpose) in order to change cognitions or emotions about performing the behavior (includes ‘Cognitive structuring’)” [43:S3].^ | Provide training to prompt nurses to deliberately adopt a new perspective on the benefits of using the EMMS as intended, for example, while it may take time to clean the COW after taking it to an isolated room, doing so **saves time** by providing easy access to information about tests and point of care information, improving legibility of medication orders. Focus on enhancing capabilities or providing other options for cleaning the COW in a time efficient way. |
|  | **Promote patient safety:** A nursing professional competency is to provide safe care, to prevent injury by identifying, eliminating or preventing environmental hazards where possible.  (Overlap with Environmental context and resources). | Take an active eMAR to the patient (B12) (B12 was also required as a part of B13, B14, B15). | [Q2] *If no - if they find out it’s too much equipment, too many furnishings in the room and it’s high risk for a fall for the patients, they can leave it outside and get the drawer. Just take the single drawer, put it on the COW and dispense the medication, put it back, check their MRN number and go to the patient and give it. (Interview 42)*  The COWs were bulky and when nurses judged that adding equipment (a COW) to already crowded rooms created a falls risk, they did not take the COW to the bedside to administer medication. | **BCT Grouping = Identity**  **BCT = Framing/reframing (*)**  “Suggest the deliberate adoption of a perspective or new perspective on behavior (e.g. its purpose) in order to change cognitions or emotions about performing the behavior (includes ‘Cognitive structuring’)” [43:S3].^ | Use persuasive communication to prompt nurses to deliberately adopt a perspective that the need to improve medication safety by taking the eMAR to the bedside and the risk of falls are not mutually exclusive – both can be achieved.  Discuss and implement strategies to decrease the furnishings in the patients’ rooms during medication administration time. |
|  | **Promote patient safety:** Assessing and managing risk to deliver safe care is an important part of a nurses’ professional role. Nurses identified the potential for interruptions and subsequent risk of medication error as a barrier to taking the COW to the patient to administer medication. A nurse’s ability to manage interruptions was associated with professional experience. |  | [Q14] *I suppose yeah I do. I get all the medications I can from the drug room, that's kind of the way I do it.* ***I always go to the drug room and try and get all the medications I can there, and then what I need to at the bedside, just because it saves me getting confused. But the more I'm at the bedside, patients start asking questions, and that's kind of when you lose your thoughts. So I'd rather look at the doses in the drug room where it's quiet, rather than at the bedside where other patients are*** *- can you come - and then you - I think most of the laptops have a sign on the back, saying not to interrupt. (Interview 91)*  Nurses prepared medications in the medication room or took bedside medication drawer to the COW in the corridor to avoid interruptions.  [Q15] *I: Once I'm in that patient's room* ***just because you've got a trolley there*** *as soon as you see a nurse it's like…*  *F: I've got my 10 things I need to ask you.*  *I: …I want 20 things in fact, I want the tissues here, I want the glass of water and the pillow's not - I need…*  *F: When's the doctor coming?*  *I: When's the doctor coming? I want to get out of here. I don't want to be here. These are the things that impede, you've got that. But that's going to happen. Once you see the nurse there - but that's not going to go away.* ***I'm very strict when I'm giving medications and I'm very assertive. A lot of the nurses probably can't formulate the language but that's just experience****. I'll say if I've got four ladies in a room and they're going I want this, I want that, I want that, I'll say, ‘Stop now’. I say, ‘I'm giving drugs, medications, unless I concentrate you get the wrong medication and I need to concentrate and it's not easy so I will get to you when I can but let me do this first’. (Nurse 03)*  When nurses took the COW to patients’ rooms to administer medication, patients interrupted them during the medication administration process. With experience (e.g. Nurse_03), nurses were able to manage the interruptions assertively. However, inexperienced nurses had not yet formulated the language to assertively manage interruptions. | **Demonstration of the behaviour**  **(*)**  “Provide an observable sample of the performance of the behaviour, directly in person or indirectly e.g. via film, pictures, for the person to aspire to or imitate (includes ‘Modelling’)” [43:S8]^ | Demonstrate to nurses how to manage interruptions during medication administration. This may be done face-to-face during group professional development sessions or may be done using a video. Allow them to practise and ask questions. |
|  | **Respect for colleagues:** The professional importance nurses placed on not impinging on their colleagues’ time was a barrier to asking a colleague to check medication during a busy time and, if required, to accompany the administering nurse to the bedside to check/witness administration. | Open a single patient’s eMAR at a time. With only their eMAR open prepare medication for one patient at a time immediately before intended use (includes medications requiring a second person check or witness) (B4). | [Q16] *Sometimes easier to get somebody to check - somebody else to* ***check them all beforehand rather than when it's six o'clock in the morning when it's a lot busier****. It won't be tomorrow.* ***Sometimes harder to disturb people - get somebody who's not doing something to just check it****. (Interview 57)* | **BCT Grouping = Identity (*)**  **BCT = Incompatible beliefs**  “Draw attention to discrepancies between current or past behavior and self-image, in order to create discomfort (includes ‘Cognitive dissonance’)” [43:S17].^ | Use persuasive communication to draw attention to the discrepancies between safe medication administration and current practice to the image of an ideal nurse. Highlight the potential implications of a medication error for the patient, the administering and the checking nurse. The mode of delivery may include a DVD or live presentation during facilitated professional development sessions. |
|  | **Respect for colleagues:** Respecting other members of the patient care team was an important part of a nurses’ professional role and identity. Nurses did not consider that logging doctors and pharmacists out of the eMAR or reclaiming the COW they had been using to administer medication (to take a COW to the bedside to administer medication) aligned with their professional identity and therefore administered medication using a desktop computer. | Take an active eMAR to the patient (B12) (B12 was also required as a part of B13, B14, B15). | [Q17] *I: Then you really want to work - that you really want to give it and then you cannot. Not like just the physical disadvantage of just moving it around but the actual medication. Only if the doctor or the pharmacist are on the same - logged in, same time as we do then whatever you give will be disappeared because they can override you. So whatever I'm doing is overridden.*  *F: So what do you do then?*  *I: Then I have to go and do it over again. I have to go all over again.*  *F: So you've got to remember to...*  *I: Yes.* ***Whereas if the doctors and the pharmacists has nurses - like when you login and say currently being used by so and so, I just exit and I wait for another five minutes. But some doctors, if it's nurse signature, nurse login they just like ah what's this...***  *F: They just override it.*  ***I: They just override it so you lose all your work****. (Interview 45)*  Nurses explained that when colleagues logged them out of the eMAR mid task, information entered but not saved to that point had to be re-entered. Nurses did not consider that it was their role/position to log doctors and pharmacists out of the eMAR. Collegiality and team work was considered an important aspect of nursing professional behaviour. Other data highlights that rather than log a colleague out or reclaim the COW they were using, nurses will use desktop computers to administer medication. Other data demonstrates that rather than loose information when logged out, nurses signed off medication in the eMAR as administered before administering it to the patient. | **Demonstration of the behaviour**  **(*)**  “Provide an observable sample of the performance of the behaviour, directly in person or indirectly e.g. via film, pictures, for the person to aspire to or imitate (includes ‘Modelling’)” [43:S8].^ | Demonstrate to nurses how to discuss with colleagues the impact of logging them off the eMAR and taking the COW when they are doing medication administration. This may be done face-to-face during group professional development sessions or may be done using a video. Allow them to practise and ask questions. |
|  | **Professional culture**: When nurses did not consider it part of their professional role to report broken equipment, there were fewer COWs available for nurses to take to the patient when administering medication. | Take an active eMAR to the patient (B12) (B12 was also required as a part of B13, B14, B15). | [Q18] *The main maintenance of the computers is one thing. We find it difficult keeping them maintained. People a) don’t take responsibility for them so won’t initiate if they notice something wrong with it, if it is physically broken or if it is a software problem they won’t initiate it, it will just be left in the corridor to the side so it decreases the number of resources” (Interview 65)*  Nurses described a professional culture in some units where reporting problems was not seen to be a collective responsibility. Rather, than report a problem with a laptop (hardware or software) so that it could be fixed, nurses left them to one side. This reduced the overall number of working COWs available for use. When there were not enough available laptops, nurses were more likely to use the desktop computers, rather than take a COW to the patient. | **Information about other’s approval (#)**  “Provide information about what other people think about the behavior. The information clarifies whether others will like, approve or disapprove of what the person is doing or will do” [43:S8]. | Provide information about what other people think about not reporting broken equipment.  Introduce posters with pictures of senior members of staff, opinion leaders and patients advocating the professional responsibility of nurses to their patients and team members to report broken equipment to ensure availability of working laptops for medication administration. Include information on how to report problems with equipment and computers on the posters. |
|  | **Professional culture** that supported senior nurses or nurses with experience not taking the COW to the bedside and hindered junior nurses from saying anything | Take an active eMAR to the patient (B12) (B12 was also required as a part of B13, B14, B15). | [Q19] *There's definitely a perception, I mean I guess there's a perception where there are even paper charts when you see a senior staff, you just think they know what they're going to do, that they know what they're doing and yeah, I think that's a lot where it stems from. You think, obviously no one's perfect, so you've got to keep checking. Yeah, and you're setting, it's out of your place, if you're younger, to say, ‘Look aren't you going to take [the COW] with you?’ (Interview 39)*  Nurses described a professional culture in which it was acceptable, particularly for senior nurses, not take the COW to the patient to administer medication. The professional hierarchy in nursing made it difficult for junior nurses to ask senior nurses to follow policy, including taking a COW to the patient. | **BCT Grouping = Identity (*)**  **BCT = Incompatible beliefs**  “Draw attention to discrepancies between current or past behavior and self-image, in order to create discomfort (includes ‘Cognitive dissonance’)” [43:S17].^ | Use persuasive communication to draw attention to the discrepancies between the potential implications of not taking the COW to the bedside to check the 5Rs (potential medication error) and the importance of safe medication administration to self-image of a professional nurse. The mode of delivery may include a DVD or live presentation during facilitated professional development sessions. |
